# Single-cell analyses and host genetics highlight the role of innate immune cells in COVID-19 severity

**DOI:** 10.1038/s41588-023-01375-1

**Published:** 2023-04-24

**Authors:** Ryuya Edahiro, Yuya Shirai, Yusuke Takeshima, Shuhei Sakakibara, Yuta Yamaguchi, Teruaki Murakami, Takayoshi Morita, Yasuhiro Kato, Yu-Chen Liu, Daisuke Motooka, Yoko Naito, Ayako Takuwa, Fuminori Sugihara, Kentaro Tanaka, James B. Wing, Kyuto Sonehara, Yoshihiko Tomofuji, Japan COVID-19 Task Force, Qingbo S. Wang, Takanori Hasegawa, Ryunosuke Saiki, Takayoshi Hyugaji, Eigo Shimizu, Kotoe Katayama, Masahiro Kanai, Tatsuhiko Naito, Noah Sasa, Kenichi Yamamoto, Kazuhisa Takahashi, Norihiro Harada, Toshio Naito, Makoto Hiki, Yasushi Matsushita, Haruhi Takagi, Masako Ichikawa, Ai Nakamura, Sonoko Harada, Yuuki Sandhu, Hiroki Kabata, Katsunori Masaki, Hirofumi Kamata, Shinnosuke Ikemura, Shotaro Chubachi, Satoshi Okamori, Hideki Terai, Atsuho Morita, Takanori Asakura, Junichi Sasaki, Hiroshi Morisaki, Yoshifumi Uwamino, Kosaku Nanki, Sho Uchida, Shunsuke Uno, Tomoyasu Nishimura, Takashi Ishiguro, Taisuke Isono, Shun Shibata, Yuma Matsui, Chiaki Hosoda, Kenji Takano, Takashi Nishida, Yoichi Kobayashi, Yotaro Takaku, Noboru Takayanagi, Soichiro Ueda, Ai Tada, Masayoshi Miyawaki, Masaomi Yamamoto, Eriko Yoshida, Reina Hayashi, Tomoki Nagasaka, Sawako Arai, Yutaro Kaneko, Kana Sasaki, Etsuko Tagaya, Masatoshi Kawana, Ken Arimura, Kunihiko Takahashi, Tatsuhiko Anzai, Satoshi Ito, Akifumi Endo, Yuji Uchimura, Yasunari Miyazaki, Takayuki Honda, Tomoya Tateishi, Shuji Tohda, Naoya Ichimura, Kazunari Sonobe, Chihiro Tani Sassa, Jun Nakajima, Yasushi Nakano, Yukiko Nakajima, Ryusuke Anan, Ryosuke Arai, Yuko Kurihara, Yuko Harada, Kazumi Nishio, Tetsuya Ueda, Masanori Azuma, Ryuichi Saito, Toshikatsu Sado, Yoshimune Miyazaki, Ryuichi Sato, Yuki Haruta, Tadao Nagasaki, Yoshinori Yasui, Yoshinori Hasegawa, Yoshikazu Mutoh, Tomoki Kimura, Tomonori Sato, Reoto Takei, Satoshi Hagimoto, Yoichiro Noguchi, Yasuhiko Yamano, Hajime Sasano, Sho Ota, Yasushi Nakamori, Kazuhisa Yoshiya, Fukuki Saito, Tomoyuki Yoshihara, Daiki Wada, Hiromu Iwamura, Syuji Kanayama, Shuhei Maruyama, Takashi Yoshiyama, Ken Ohta, Hiroyuki Kokuto, Hideo Ogata, Yoshiaki Tanaka, Kenichi Arakawa, Masafumi Shimoda, Takeshi Osawa, Hiroki Tateno, Isano Hase, Shuichi Yoshida, Shoji Suzuki, Miki Kawada, Hirohisa Horinouchi, Fumitake Saito, Keiko Mitamura, Masao Hagihara, Junichi Ochi, Tomoyuki Uchida, Rie Baba, Daisuke Arai, Takayuki Ogura, Hidenori Takahashi, Shigehiro Hagiwara, Genta Nagao, Shunichiro Konishi, Ichiro Nakachi, Koji Murakami, Mitsuhiro Yamada, Hisatoshi Sugiura, Hirohito Sano, Shuichiro Matsumoto, Nozomu Kimura, Yoshinao Ono, Hiroaki Baba, Yusuke Suzuki, Sohei Nakayama, Keita Masuzawa, Shinichi Namba, Takayuki Shiroyama, Yoshimi Noda, Takayuki Niitsu, Yuichi Adachi, Takatoshi Enomoto, Saori Amiya, Reina Hara, Tomoki Kuge, Kinnosuke Matsumoto, Yuji Yamamoto, Makoto Yamamoto, Midori Yoneda, Kazunori Tomono, Kazuto Kato, Hidefumi Koh, Tadashi Manabe, Yohei Funatsu, Fumimaro Ito, Takahiro Fukui, Keisuke Shinozuka, Sumiko Kohashi, Masatoshi Miyazaki, Tomohisa Shoko, Mitsuaki Kojima, Tomohiro Adachi, Motonao Ishikawa, Kenichiro Takahashi, Takashi Inoue, Toshiyuki Hirano, Keigo Kobayashi, Hatsuyo Takaoka, Kazuyoshi Watanabe, Naoki Miyazawa, Yasuhiro Kimura, Reiko Sado, Hideyasu Sugimoto, Akane Kamiya, Naota Kuwahara, Akiko Fujiwara, Tomohiro Matsunaga, Yoko Sato, Takenori Okada, Yoshihiro Hirai, Hidetoshi Kawashima, Atsuya Narita, Kazuki Niwa, Yoshiyuki Sekikawa, Koichi Nishi, Masaru Nishitsuji, Mayuko Tani, Junya Suzuki, Hiroki Nakatsumi, Takashi Ogura, Hideya Kitamura, Eri Hagiwara, Kota Murohashi, Hiroko Okabayashi, Takao Mochimaru, Shigenari Nukaga, Ryosuke Satomi, Yoshitaka Oyamada, Nobuaki Mori, Tomoya Baba, Yasutaka Fukui, Mitsuru Odate, Shuko Mashimo, Yasushi Makino, Kazuma Yagi, Mizuha Hashiguchi, Junko Kagyo, Tetsuya Shiomi, Satoshi Fuke, Hiroshi Saito, Tomoya Tsuchida, Shigeki Fujitani, Mumon Takita, Daiki Morikawa, Toru Yoshida, Takehiro Izumo, Minoru Inomata, Naoyuki Kuse, Nobuyasu Awano, Mari Tone, Akihiro Ito, Yoshihiko Nakamura, Kota Hoshino, Junichi Maruyama, Hiroyasu Ishikura, Tohru Takata, Toshio Odani, Masaru Amishima, Takeshi Hattori, Yasuo Shichinohe, Takashi Kagaya, Toshiyuki Kita, Kazuhide Ohta, Satoru Sakagami, Kiyoshi Koshida, Kentaro Hayashi, Tetsuo Shimizu, Yutaka Kozu, Hisato Hiranuma, Yasuhiro Gon, Namiki Izumi, Kaoru Nagata, Ken Ueda, Reiko Taki, Satoko Hanada, Kodai Kawamura, Kazuya Ichikado, Kenta Nishiyama, Hiroyuki Muranaka, Kazunori Nakamura, Naozumi Hashimoto, Keiko Wakahara, Sakamoto Koji, Norihito Omote, Akira Ando, Nobuhiro Kodama, Yasunari Kaneyama, Shunsuke Maeda, Takashige Kuraki, Takemasa Matsumoto, Koutaro Yokote, Taka-Aki Nakada, Ryuzo Abe, Taku Oshima, Tadanaga Shimada, Masahiro Harada, Takeshi Takahashi, Hiroshi Ono, Toshihiro Sakurai, Takayuki Shibusawa, Yoshifumi Kimizuka, Akihiko Kawana, Tomoya Sano, Chie Watanabe, Ryohei Suematsu, Hisako Sageshima, Ayumi Yoshifuji, Kazuto Ito, Saeko Takahashi, Kota Ishioka, Morio Nakamura, Makoto Masuda, Aya Wakabayashi, Hiroki Watanabe, Suguru Ueda, Masanori Nishikawa, Yusuke Chihara, Mayumi Takeuchi, Keisuke Onoi, Jun Shinozuka, Atsushi Sueyoshi, Yoji Nagasaki, Masaki Okamoto, Sayoko Ishihara, Masatoshi Shimo, Yoshihisa Tokunaga, Yu Kusaka, Takehiko Ohba, Susumu Isogai, Aki Ogawa, Takuya Inoue, Satoru Fukuyama, Yoshihiro Eriguchi, Akiko Yonekawa, Keiko Kan-o, Koichiro Matsumoto, Kensuke Kanaoka, Shoichi Ihara, Kiyoshi Komuta, Yoshiaki Inoue, Shigeru Chiba, Kunihiro Yamagata, Yuji Hiramatsu, Hirayasu Kai, Koichiro Asano, Tsuyoshi Oguma, Yoko Ito, Satoru Hashimoto, Masaki Yamasaki, Yu Kasamatsu, Yuko Komase, Naoya Hida, Takahiro Tsuburai, Baku Oyama, Minoru Takada, Hidenori Kanda, Yuichiro Kitagawa, Tetsuya Fukuta, Takahito Miyake, Shozo Yoshida, Shinji Ogura, Shinji Abe, Yuta Kono, Yuki Togashi, Hiroyuki Takoi, Ryota Kikuchi, Shinichi Ogawa, Tomouki Ogata, Shoichiro Ishihara, Arihiko Kanehiro, Shinji Ozaki, Yasuko Fuchimoto, Sae Wada, Nobukazu Fujimoto, Kei Nishiyama, Mariko Terashima, Satoru Beppu, Kosuke Yoshida, Osamu Narumoto, Hideaki Nagai, Nobuharu Ooshima, Mitsuru Motegi, Akira Umeda, Kazuya Miyagawa, Hisato Shimada, Mayu Endo, Yoshiyuki Ohira, Masafumi Watanabe, Sumito Inoue, Akira Igarashi, Masamichi Sato, Hironori Sagara, Akihiko Tanaka, Shin Ohta, Tomoyuki Kimura, Yoko Shibata, Yoshinori Tanino, Takefumi Nikaido, Hiroyuki Minemura, Yuki Sato, Yuichiro Yamada, Takuya Hashino, Masato Shinoki, Hajime Iwagoe, Hiroshi Takahashi, Kazuhiko Fujii, Hiroto Kishi, Masayuki Kanai, Tomonori Imamura, Tatsuya Yamashita, Masakiyo Yatomi, Toshitaka Maeno, Shinichi Hayashi, Mai Takahashi, Mizuki Kuramochi, Isamu Kamimaki, Yoshiteru Tominaga, Tomoo Ishii, Mitsuyoshi Utsugi, Akihiro Ono, Toru Tanaka, Takeru Kashiwada, Kazue Fujita, Yoshinobu Saito, Masahiro Seike, Hiroko Watanabe, Hiroto Matsuse, Norio Kodaka, Chihiro Nakano, Takeshi Oshio, Takatomo Hirouchi, Shohei Makino, Moritoki Egi, Yosuke Omae, Yasuhito Nannya, Takafumi Ueno, Tomomi Takano, Kazuhiko Katayama, Masumi Ai, Toshiro Sato, Naoki Hasegawa, Katsushi Tokunaga, Makoto Ishii, Ryuji Koike, Yuko Kitagawa, Akinori Kimura, Seiya Imoto, Satoru Miyano, Seishi Ogawa, Takanori Kanai, Ho Namkoong, Hiromu Tanaka, Ho Lee, Koichi Fukunaga, Haruhiko Hirata, Yoshito Takeda, Daisuke Okuzaki, Atsushi Kumanogoh, Yukinori Okada

**Affiliations:** 1 Department of Statistical Genetics, Osaka University Graduate School of Medicine, Suita, Japan; 2 Department of Respiratory Medicine and Clinical Immunology, Osaka University Graduate School of Medicine, Suita, Japan; 3 Laboratory of Statistical Immunology, Immunology Frontier Research Center (WPI-IFReC), Osaka University, Suita, Japan; 4 Laboratory of Experimental Immunology, Immunology Frontier Research Center (WPI-IFReC), Osaka University, Suita, Japan; 5 Laboratory of Immune Regulation, Immunology Frontier Research Center, Osaka University, Suita, Japan; 6 Department of Immunopathology, Immunology Frontier Research Center (WPI-IFReC), Osaka University, Suita, Japan; 7 Laboratory of Human Immunology (Single Cell Genomics), WPI Immunology Frontier Research Center, Osaka University, Suita, Japan; 8 Genome Information Research Center, Research Institute for Microbial Diseases, Osaka University, Suita, Japan; 9 Integrated Frontier Research for Medical Science Division, Institute for Open and Transdisciplinary Research Initiatives, Osaka University, Suita, Japan; 10 Core Instrumentation Facility, Immunology Frontier Research Center and Research Institute for Microbial Diseases, Osaka University, Suita, Japan; 11 Laboratory of Human Immunology (Single Cell Immunology), Immunology Frontier Research Center, Osaka University, Suita, Japan; 12 Center for Infectious Disease Education and Research (CiDER), Osaka University, Suita, Japan; 13 Laboratory for Systems Genetics, RIKEN Center for Integrative Medical Sciences, Yokohama, Japan; 14 Department of Genome Informatics, Graduate School of Medicine, The University of Tokyo, Tokyo, Japan; 15 Department of Infectious Diseases, Keio University School of Medicine, Tokyo, Japan; 16 Division of Pulmonary Medicine, Department of Medicine, Keio University School of Medicine, Tokyo, Japan; 17 Japan Agency for Medical Research and Development – Core Research for Evolutional Science and Technology (AMED–CREST), Osaka University, Osaka, Japan; 18 M&D Data Science Center, Tokyo Medical and Dental University, Tokyo, Japan; 19 Department of Pathology and Tumor Biology, Kyoto University, Kyoto, Japan; 20 Division of Health Medical Intelligence, Human Genome Center, the Institute of Medical Science, The University of Tokyo, Tokyo, Japan; 21 Department of Biomedical Informatics, Harvard Medical School, Boston, MA USA; 22 Department of Otorhinolaryngology—Head and Neck Surgery, Osaka University Graduate School of Medicine, Suita, Japan; 23 Department of Pediatrics, Osaka University Graduate School of Medicine, Suita, Japan; 24 Department of Respiratory Medicine, Juntendo University Faculty of Medicine and Graduate School of Medicine, Tokyo, Japan; 25 Department of General Medicine, Juntendo University Faculty of Medicine and Graduate School of Medicine, Tokyo, Japan; 26 Department of Emergency and Disaster Medicine, Juntendo University Faculty of Medicine and Graduate School of Medicine, Tokyo, Japan; 27 Department of Cardiovascular Biology and Medicine, Juntendo University Faculty of Medicine and Graduate School of Medicine, Tokyo, Japan; 28 Department of Internal Medicine and Rheumatology, Juntendo University Faculty of Medicine and Graduate School of Medicine, Tokyo, Japan; 29 Atopy (Allergy) Research Center, Juntendo University Graduate School of Medicine, Tokyo, Japan; 30 Department of Emergency and Critical Care Medicine, Keio University School of Medicine, Tokyo, Japan; 31 Department of Anesthesiology, Keio University School of Medicine, Tokyo, Japan; 32 Department of Laboratory Medicine, Keio University School of Medicine, Tokyo, Japan; 33 Division of Gastroenterology and Hepatology, Department of Medicine Keio University School of Medicine, Tokyo, Japan; 34 Keio University Health Center, Tokyo, Japan; 35 Department of Respiratory Medicine, Saitama Cardiovascular and Respiratory Center, Kumagaya, Japan; 36 Department of Internal Medicine, Japan Community Health Care Organization (JCHO) Saitama Medical Center, Saitama, Japan; 37 Department of Respiratory Medicine, Tokyo Women’s Medical University, Tokyo, Japan; 38 Department of General Medicine, Tokyo Women’s Medical University, Tokyo, Japan; 39 Clinical Research Center, Tokyo Medical and Dental University Hospital of Medicine, Tokyo, Japan; 40 Department of Medical Informatics, Tokyo Medical and Dental University Hospital of Medicine, Tokyo, Japan; 41 Respiratory Medicine, Tokyo Medical and Dental University, Tokyo, Japan; 42 Clinical Laboratory, Tokyo Medical and Dental University Hospital of Medicine, Tokyo, Japan; 43 Kawasaki Municipal Ida Hospital, Department of Internal Medicine, Kawasaki, Japan; 44 Department of Respiratory Medicine, Osaka Saiseikai Nakatsu Hospital, Osaka, Japan; 45 Department of Infection Control, Osaka Saiseikai Nakatsu Hospital, Osaka, Japan; 46 Department of Infectious Diseases, Tosei General Hospital, Seto, Japan; 47 Department of Respiratory Medicine and Allergy, Tosei General Hospital, Seto, Japan; 48 Department of Emergency and Critical Care Medicine, Kansai Medical University General Medical Center, Moriguchi, Japan; 49 Japan Anti-Tuberculosis Association (JATA) Fukujuji Hospital, Kiyose, Japan; 50 Department of Pulmonary Medicine, Saitama City Hospital, Saitama, Japan; 51 Department of Infectious Diseases, Saitama City Hospital, Saitama, Japan; 52 Department of General Thoracic Surgery, Saitama City Hospital, Saitama, Japan; 53 Department of Pulmonary Medicine, Eiju General Hospital, Tokyo, Japan; 54 Division of Infection Control, Eiju General Hospital, Tokyo, Japan; 55 Department of Hematology, Eiju General Hospital, Tokyo, Japan; 56 Saiseikai Utsunomiya Hospital, Utsunomiya, Japan; 57 Department of Respiratory Medicine, Tohoku University Graduate School of Medicine, Sendai, Japan; 58 Department of Infectious Diseases, Tohoku University Graduate School of Medicine, Sendai, Japan; 59 Department of Respiratory Medicine, Kitasato University Kitasato Institute Hospital, Tokyo, Japan; 60 Division of Infection Control and Prevention, Osaka University Hospital, Suita, Japan; 61 Department of Biomedical Ethics and Public Policy, Osaka University Graduate School of Medicine, Suita, Japan; 62 Tachikawa Hospital, Tachikawa, Japan; 63 Department of Emergency and Critical Care Medicine, Tokyo Women’s Medical University Medical Center East, Tokyo, Japan; 64 Department of Medicine, Tokyo Women’s Medical University Medical Center East, Tokyo, Japan; 65 Department of Pediatrics, Tokyo Women’s Medical University Medical Center East, Tokyo, Japan; 66 Department of Internal Medicine, Sano Kosei General Hospital, Sano, Japan; 67 Japan Community Health care Organization Kanazawa Hospital, Kanazawa, Japan; 68 Department of Respiratory Medicine, Saiseikai Yokohamashi Nanbu Hospital, Yokohama, Japan; 69 Department of Clinical Laboratory, Saiseikai Yokohamashi Nanbu Hospital, Yokohama, Japan; 70 Department of Internal Medicine, Internal Medicine Center, Showa University Koto Toyosu Hospital, Tokyo, Japan; 71 Department of Respiratory Medicine, Japan Organization of Occupational Health and Safety, Kanto Rosai Hospital, Kawasaki, Japan; 72 Department of General Internal Medicine, Japan Organization of Occupational Health and Safety, Kanto Rosai Hospital, Kawasaki, Japan; 73 Ishikawa Prefectural Central Hospital, Kanazawa, Japan; 74 Kanagawa Cardiovascular and Respiratory Center, Yokohama, Japan; 75 Department of Respiratory Medicine, National Hospital Organization Tokyo Medical Center, Tokyo, Japan; 76 Department of Allergy, National Hospital Organization Tokyo Medical Center, Tokyo, Japan; 77 Department of General Internal Medicine and Infectious Diseases, National Hospital Organization Tokyo Medical Center, Tokyo, Japan; 78 Department of Respiratory Medicine, Toyohashi Municipal Hospital, Toyohashi, Japan; 79 Keiyu Hospital, Yokohama, Japan; 80 Department of Respiratory Medicine, KKR Sapporo Medical Center, Sapporo, Japan; 81 Division of General Internal Medicine, Department of Internal Medicine, St. Marianna University School of Medicine, Kawasaki, Japan; 82 Department of Emergency and Critical Care Medicine, St. Marianna University School of Medicine, Kawasaki, Japan; 83 Japanese Red Cross Medical Center, Tokyo, Japan; 84 Matsumoto City Hospital, Matsumoto, Japan; 85 Department of Emergency and Critical Care Medicine, Faculty of Medicine, Fukuoka University, Fukuoka, Japan; 86 Department of Infection Control, Fukuoka University Hospital, Fukuoka, Japan; 87 Department of Rheumatology, National Hospital Organization Hokkaido Medical Center, Sapporo, Japan; 88 Department of Respiratory Medicine, National Hospital Organization Hokkaido Medical Center, Sapporo, Japan; 89 Department of Emergency and Critical Care Medicine, National Hospital Organization Hokkaido Medical Center, Sapporo, Japan; 90 National Hospital Organization Kanazawa Medical Center, Kanazawa, Japan; 91 Nihon University School of Medicine, Department of Internal Medicine, Division of Respiratory Medicine, Tokyo, Japan; 92 Musashino Red Cross Hospital, Musashino, Japan; 93 Division of Respiratory Medicine, Social Welfare Organization Saiseikai Imperial Gift Foundation, Inc., Saiseikai Kumamoto Hospital, Kumamoto, Japan; 94 Department of Respiratory Medicine, Nagoya University Graduate School of Medicine, Nagoya, Japan; 95 Fukuoka Tokushukai Hospital, Department of Internal Medicine, Kasuga, Japan; 96 Fukuoka Tokushukai Hospital, Respiratory Medicine, Kasuga, Japan; 97 Department of Endocrinology, Hematology and Gerontology, Chiba University Graduate School of Medicine, Chiba, Japan; 98 Department of Emergency and Critical Care Medicine, Chiba University Graduate School of Medicine, Chiba, Japan; 99 National Hospital Organization Kumamoto Medical Center, Kumamoto, Japan; 100 Division of Infectious Diseases and Respiratory Medicine, Department of Internal Medicine, National Defense Medical College, Tokorozawa, Japan; 101 Sapporo City General Hospital, Sapporo, Japan; 102 Department of Internal Medicine, Tokyo Saiseikai Central Hospital, Tokyo, Japan; 103 Department of Pulmonary Medicine, Tokyo Saiseikai Central Hospital, Tokyo, Japan; 104 Department of Respiratory Medicine, Fujisawa City Hospital, Fujisawa, Japan; 105 Uji-Tokushukai Medical Center, Uji, Japan; 106 Department of Infectious Disease and Clinical Research Institute, National Hospital Organization Kyushu Medical Center, Fukuoka, Japan; 107 Department of Respirology, National Hospital Organization Kyushu Medical Center, Fukuoka, Japan; 108 Division of Respirology, Rheumatology, and Neurology, Department of Internal Medicine, Kurume University School of Medicine, Kurume, Japan; 109 Department of Infectious Disease, National Hospital Organization Kyushu Medical Center, Fukuoka, Japan; 110 Ome Municipal General Hospital, Ome, Japan; 111 Research Institute for Diseases of the Chest, Graduate School of Medical Sciences, Kyushu University, Fukuoka, Japan; 112 Department of Medicine and Biosystemic Science, Kyushu University Graduate School of Medical Sciences, Fukuoka, Japan; 113 Daini Osaka Police Hospital, Osaka, Japan; 114 Department of Emergency and Critical Care Medicine, Faculty of Medicine, University of Tsukuba, Tsukuba, Japan; 115 Department of Hematology, Faculty of Medicine, University of Tsukuba, Tsukuba, Japan; 116 Department of Nephrology, Faculty of Medicine, University of Tsukuba, Tsukuba, Japan; 117 Department of Cardiovascular Surgery, Faculty of Medicine, University of Tsukuba, Tsukuba, Japan; 118 Division of Pulmonary Medicine, Department of Medicine, Tokai University School of Medicine, Isehara, Japan; 119 Department of Anesthesiology and Intensive Care Medicine, Kyoto Prefectural University of Medicine, Kyoto, Japan; 120 Department of Infection Control and Laboratory Medicine, Kyoto Prefectural University of Medicine, Kyoto, Japan; 121 Department of Respiratory Internal Medicine, St. Marianna University School of Medicine, Yokohama-City Seibu Hospital, Yokohama, Japan; 122 KINSHUKAI Hanwa The Second Hospital, Osaka, Japan; 123 Emergency and Disaster Medicine, Gifu University School of Medicine Graduate School of Medicine, Gifu, Japan; 124 Department of Respiratory Medicine, Tokyo Medical University Hospital, Tokyo, Japan; 125 JA Toride Medical Hospital, Toride, Japan; 126 Okayama Rosai Hospital, Okayama, Japan; 127 Himeji St. Mary’s Hospital, Himeji, Japan; 128 Emergency & Critical Care, Niigata University, Niigata, Japan; 129 Emergency & Critical Care Center, National Hospital Organization Kyoto Medical Center, Kyoto, Japan; 130 National Hospital Organization Tokyo Hospital Hospital, Kiyose, Japan; 131 Fujioka General Hospital, Fujioka, Japan; 132 Department of General Medicine, School of Medicine, International University of Health and Welfare Shioya Hospital, Ohtawara, Japan; 133 Department of Pharmacology, School of Pharmacy, International University of Health and Welfare Shioya Hospital, Ohtawara, Japan; 134 Department of Respiratory Medicine, International University of Health and Welfare Shioya Hospital, Ohtawara, Japan; 135 Department of Clinical Laboratory, International University of Health and Welfare Shioya Hospital, Ohtawara, Japan; 136 Department of Cardiology, Pulmonology, and Nephrology, Yamagata University Faculty of Medicine, Yamagata, Japan; 137 Division of Respiratory Medicine and Allergology, Department of Medicine, School of Medicine, Showa University, Tokyo, Japan; 138 Department of Pulmonary Medicine, Fukushima Medical University, Fukushima, Japan; 139 Kansai Electric Power Hospital, Osaka, Japan; 140 Division of Infectious Diseases, Kumamoto City Hospital, Kumamoto, Japan; 141 Department of Respiratory Medicine, Kumamoto City Hospital, Kumamoto, Japan; 142 Department of Emergency and Critical Care Medicine, Tokyo Metropolitan Police Hospital, Tokyo, Japan; 143 Department of Respiratory Medicine, Gunma University Graduate School of Medicine, Maebashi, Japan; 144 National Hospital Organization Saitama Hospital, Wako, Japan; 145 Tokyo Medical University Ibaraki Medical Center, Inashiki, Japan; 146 Department of Internal Medicine, Kiryu Kosei General Hospital, Kiryu, Japan; 147 Department of Pulmonary Medicine and Oncology, Graduate School of Medicine, Nippon Medical School, Tokyo, Japan; 148 Division of Respiratory Medicine, Tsukuba Kinen General Hospital, Tsukuba, Japan; 149 Division of Respiratory Medicine, Department of Internal Medicine, Toho University Ohashi Medical Center, Tokyo, Japan; 150 Division of Anesthesiology, Department of Surgery, Kobe University Graduate School of Medicine, Kobe, Japan; 151 Genome Medical Science Project (Toyama), National Center for Global Health and Medicine, Tokyo, Japan; 152 Department of Biomolecular Engineering, Graduate School of Tokyo Institute of Technology, Tokyo, Japan; 153 Laboratory of Veterinary Infectious Disease, School of Veterinary Medicine, Kitasato University, Aomori, Japan; 154 Laboratory of Viral Infection, Department of Infection Control and Immunology, Ōmura Satoshi Memorial Institute & Graduate School of Infection Control Sciences, Kitasato University, Tokyo, Japan; 155 Department of Insured Medical Care Management, Tokyo Medical and Dental University Hospital of Medicine, Tokyo, Japan; 156 Department of Organoid Medicine, Keio University School of Medicine, Tokyo, Japan; 157 Medical Innovation Promotion Center, Tokyo Medical and Dental University, Tokyo, Japan; 158 Department of Surgery, Keio University School of Medicine, Tokyo, Japan; 159 Institute of Research, Tokyo Medical and Dental University, Tokyo, Japan; 160 Institute for the Advanced Study of Human Biology (WPI-ASHBi), Kyoto University, Kyoto, Japan; 161 Department of Medicine, Center for Hematology and Regenerative Medicine, Karolinska Institute, Stockholm, Sweden; 162 AMED-CREST, Japan Agency for Medical Research and Development, Tokyo, Japan

**Keywords:** Gene expression, Infectious diseases, Gene expression profiling

## Abstract

Mechanisms underpinning the dysfunctional immune response in severe acute respiratory syndrome coronavirus 2 infection are elusive. We analyzed single-cell transcriptomes and T and B cell receptors (BCR) of >895,000 peripheral blood mononuclear cells from 73 coronavirus disease 2019 (COVID-19) patients and 75 healthy controls of Japanese ancestry with host genetic data. COVID-19 patients showed a low fraction of nonclassical monocytes (ncMono). We report downregulated cell transitions from classical monocytes to ncMono in COVID-19 with reduced *CXCL10* expression in ncMono in severe disease. Cell–cell communication analysis inferred decreased cellular interactions involving ncMono in severe COVID-19. Clonal expansions of BCR were evident in the plasmablasts of patients. Putative disease genes identified by COVID-19 genome-wide association study showed cell type-specific expressions in monocytes and dendritic cells. A COVID-19-associated risk variant at the *IFNAR2* locus (rs13050728) had context-specific and monocyte-specific expression quantitative trait loci effects. Our study highlights biological and host genetic involvement of innate immune cells in COVID-19 severity.

## Abstract (web summary)

Peripheral blood mononuclear cells from 73 Japanese patients with coronavirus disease 2019 (COVID-19) and 75 healthy controls were analyzed using single-cell transcriptomics. Combining these data with genotyping data highlights the interplay between host genetics and the immune response in modulating disease severity.

## Main

Coronavirus disease 2019 (COVID-19) caused by severe acute respiratory syndrome coronavirus 2 (SARS-CoV-2) represents a serious global public health issue^1^. The clinical presentation of COVID-19 is highly variable, ranging from asymptomatic infection to fatal respiratory/multi-organ failure^2^. Although effective vaccines have successfully reduced both viral transmission and disease burden^3,4^, there exists an urgent need to elucidate the mechanism of severe COVID-19 to predict its severity and develop new treatments.

Multiple studies have highlighted dysregulation of complex networks of peripheral blood immune responses in COVID-19, using single-cell RNA-sequencing (scRNA-seq) analysis^5–14^. Monocytes^5–9^, antigen-presenting cells^10^, natural killer (NK) cells^5,6,11^, T cells^5–7,12^ and B cells^5–7^ are all reported to be related to the severity of COVID-19, while a dysregulated interferon (IFN) response^8,14,15^, which has a key role on innate immune response^16^, is closely associated with the pathogenesis of COVID-19 severity. Although these studies give us important aspects of the immunopathology of COVID-19, the immune response of the host to SARS-CoV-2 still remains unclear.

In addition, genome-wide association studies (GWASs) of COVID-19, highlighted as an achievement by the COVID-19 host genetics initiative (HGI), have demonstrated that the host genetic backgrounds influence susceptibility to and/or severity of COVID-19 (refs. ^17–19^). Multiple genetic variants associated with the COVID-19 risk are shared across different populations, while population-specific risk variants also have been reported^20,21^. Considering the different prognoses by ancestry^22^, integrated analysis of transcriptome and genetic data at single-cell resolution by ancestry should provide new insights.

Here we performed a detailed scRNA-seq analysis of over 895,000 peripheral blood mononuclear cells (PBMCs) of 73 patients with COVID-19 as well as 75 healthy controls of Japanese ancestry, and then context-specific and cell type-specific expression quantitative trait loci (eQTL) analysis by integrating scRNA-seq and host genetics data.

The proportion of nonclassical monocytes (ncMono) decreased in COVID-19 patients and RNA velocity analysis revealed the downregulation of the cellular transitions from classical monocytes (cMono) to ncMono in COVID-19 patients. We found that *CXCL10* expression was downregulated in ncMono during severe COVID-19, and cell–cell communication analysis inferred that the cellular interactions involving ncMono and plasmacytoid dendritic cells (pDC) were reduced in severe COVID-19. The putative disease genes identified by the GWAS of severe COVID-19 were enriched in monocytes and dendritic cells (DC), and COVID-19-associated variants had context and cell type-specific eQTL effects, with the *IFNAR2* variant (rs13050728) in particular having COVID-19-specific and monocytes-specific eQTL effect. In summary, our data linked innate immune cell dysfunction, especially ncMono, with severe COVID-19 and demonstrated the enrichment of host genetic risk in innate immune cells, indicating biological and host genetic critical involvement of innate immune cells in COVID-19 severity.

## Results

### Single-cell transcriptional profiling of COVID-19 PBMC

**Fig. 1:**
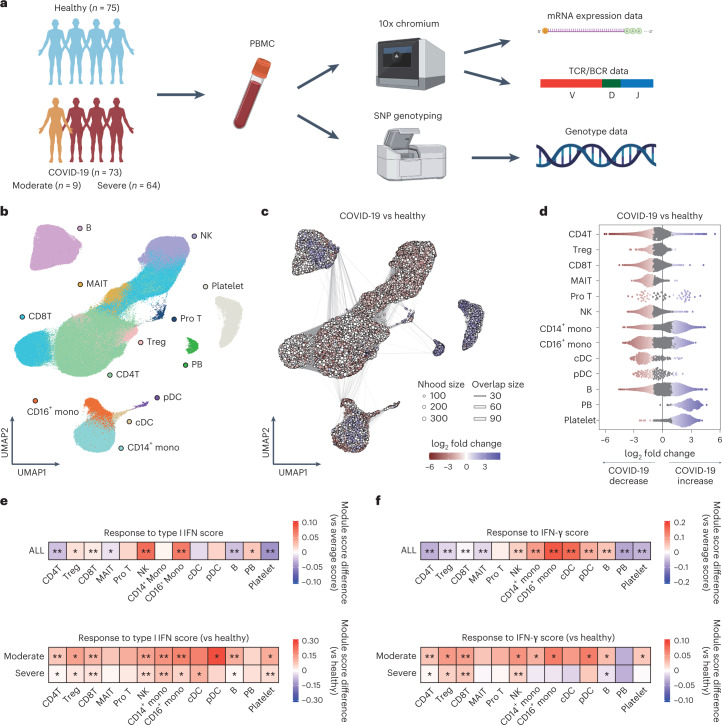
Study design and single-cell transcriptional analysis of PBMCs from COVID-19 patients and healthy controls. **a**, Overview of the study design. The image was created using BioRender.com. **b**, UMAP embedding of scRNA-seq data for all 895,460 cells. Thirteen cell types were defined by RNA expression of marker genes (Extended Data Fig. 1a). **c**, Graph representation of Nhoods identified by Milo. Nodes are Nhoods, colored by their log_2_ FC between COVID-19 patients (*n* = 72) and healthy controls (*n* = 75) adjusted by age and sex. Nondifferential abundance Nhoods (FDR ≥ 0.1) are colored white, and sizes correspond to the number of cells in a Nhood. Graph edges depict the number of cells shared between adjacent Nhoods. **d**, Beeswarm plot showing the distribution of adjusted log_2_ FC in abundance between COVID-19 patients and healthy controls in Nhoods according to 13 cell types. Colors are represented in the same way as in **c**. **e**,**f**, The module score of Type I IFN response and IFN-γ response in PBMCs. The score was calculated using a gene set termed ‘GOBP_RESPONSE_TO_TYPE_I_INTERFERON’ (GO:0034340) and ‘GOBP_RESPONSE_TO_INTERFERON_GAMMA’ (GO:0034341), respectively. Upper heatmaps depicting the difference between average scores of 13 cell types and that of all single cells. The module scores of cells in each cell type were compared with the average score of all PBMCs using a two-sided one-sample *t*-test. Lower heatmaps depicting the difference between average scores of moderate or severe disease group and those of the healthy group in each of 13 cell types (*n* = 75 healthy, *n* = 9 moderate, *n* = 64 severe). The module scores of cells of moderate or severe disease group were compared to those of healthy group in each cell type using a two-sided Welch’s *t*-test. *P_uncorrected_ < 1.0 × 10^−50^, **P_uncorrected_ < 1.0 × 10^−300^. Mono, monocytes; Pro, proliferative; Nhood, neighborhood.

To investigate the immunopathogenesis and host genetics mechanism of SARS-CoV-2 infections in COVID-19 patients, PBMCs were collected from 73 COVID-19 patients and 75 healthy controls of Japanese ancestry at Osaka University. The 73 patients with COVID-19 were classified into two conditions as follows: moderate (*n* = 9) and severe (*n* = 64) disease according to the WHO classification^23^ (Fig. 1a and Supplementary Table 1). No significant difference was noted in age distribution and sex composition between moderate and severe disease groups. The clinical characteristics are summarized in Supplementary Table 1.

**Fig. 2:**
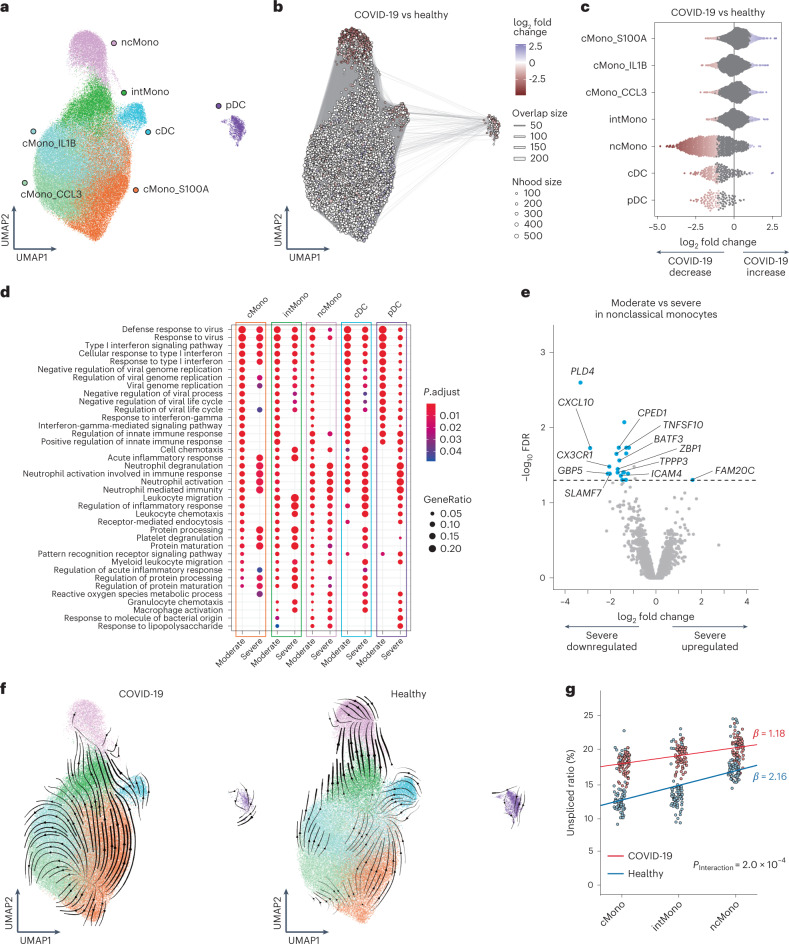
Defective IFN-γ response and reduced transition potential to ncMono in monocytes of severe COVID-19. **a**, UMAP embedding of 116,944 monocytes and DC. Seven cell types were defined by RNA expression of marker genes (Extended Data Fig. 2a). **b**, Graph representation of Nhoods identified by Milo. Nodes are Nhoods, colored by their log_2_ FC between COVID-19 (*n* = 72) and healthy controls (*n* = 75) adjusted by age and sex. Nondifferential abundance Nhoods (FDR ≥ 0.1) are colored white. **c**, Beeswarm plot showing the distribution of adjusted log_2_ FC in abundance between COVID-19 and healthy controls in Nhoods according to seven cell types. Colors are represented in the same way as in **b**. **d**, The top ten enriched biological processes by GO analysis of upregulated DEGs of moderate and severe disease compared to healthy group in five cell types. Dot color indicates the statistical significance of the enrichment (adjusted *P* values via the Benjamini–Hochberg method), and dot size represents gene ratio annotated to each term. **e**, The differential gene expression analysis between moderate (*n* = 8) and severe (*n* = 64) COVID-19 in ncMono. DEGs (FDR < 0.05 and FC > 2) are colored in light blue and labeled by gene symbols if log_2_ FC > 1.5. **f**, Velocities derived from the dynamical model for monocytes and DC cluster from COVID-19 and healthy group are projected into a UMAP-based embedding. Colors indicate the same clusters as in **a**. **g**, Average unspliced ratio of each sample stratified by three monocytes clusters, colored by COVID-19 (*n* = 73) and healthy (*n* = 75) groups. Condition-specific regression lines are shown. *P* value for the interaction effect between three monocyte clusters and two clinical conditions is uncorrected and reflects two-sided test.

**Fig. 3:**
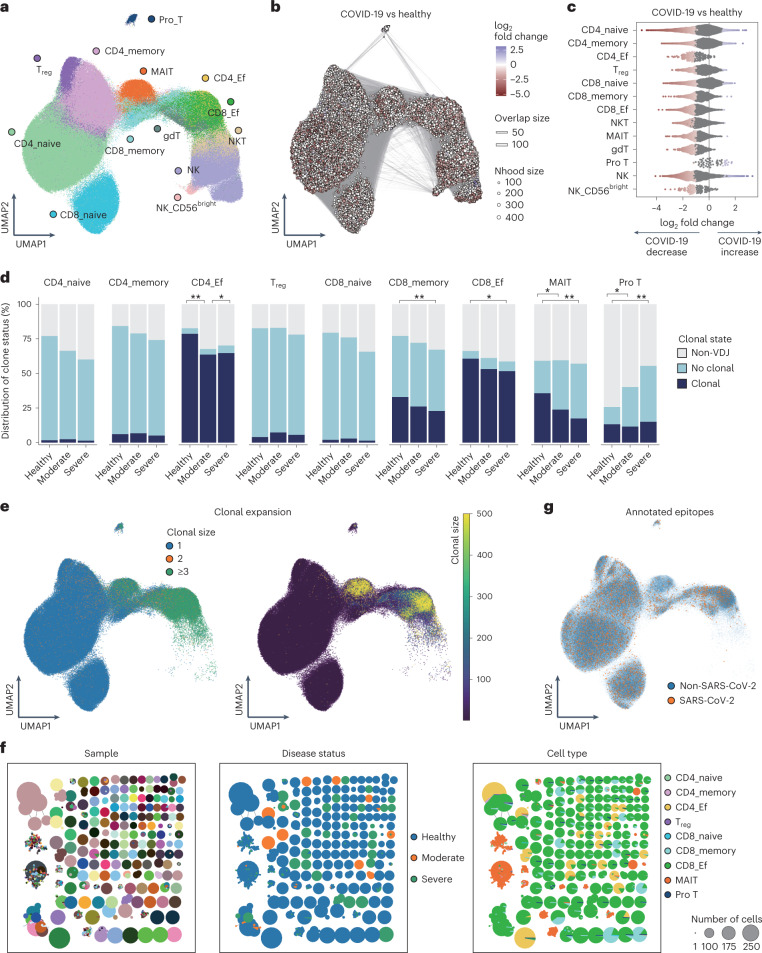
Differential abundance analysis of T cells and NK cells and TCR analysis. **a**, UMAP embedding of 628,715 T and NK cells. Thirteen cell types were defined by RNA expression of marker genes (Extended Data Fig. 3a). **b**, Graph representation of Nhoods identified by Milo. Nodes are Nhoods, colored by their log_2_ FC between COVID-19 (*n* = 72) and healthy controls (*n* = 75) adjusted by age and sex. Nondifferential abundance Nhoods (FDR ≥ 0.1) are colored white. **c**, Beeswarm plot showing the distribution of adjusted log_2_ FC in abundance between COVID-19 and healthy controls in Nhoods according to 13 cell types. Colors are represented in the same way as in **b**. **d**, The distribution of the clone state of T cells in each cluster according to disease status. Differences of average clonal expansion rate of each sample between clinical conditions were evaluated in each cluster using two-sided Welch’s *t*-test (*P_uncorrected_ < 0.05, **P_uncorrected_ < 0.005), where only cells mapped with TCRs were included in the analysis (*n* = 75 healthy, *n* = 9 moderate, *n* = 64 severe). **e**, UMAP embedding of T cells (nine cell types) colored by clonal expansion size. Left panel shows clonal expansion divided into three categories, and right panel shows clonal expansion sizes ranging from 0 to 500. **f**, Network plots showing similarity of TCRα and TCRβ CDR3 amino acid sequence for each sample, disease status and cell types. Clonotype clusters with clonal size ≥50 are selected. **g**, T cells that were suspected to be specific to SARS-CoV-2 based on CDR3 amino acid sequence were projected on UMAP. Ef, effector.

**Fig. 4:**
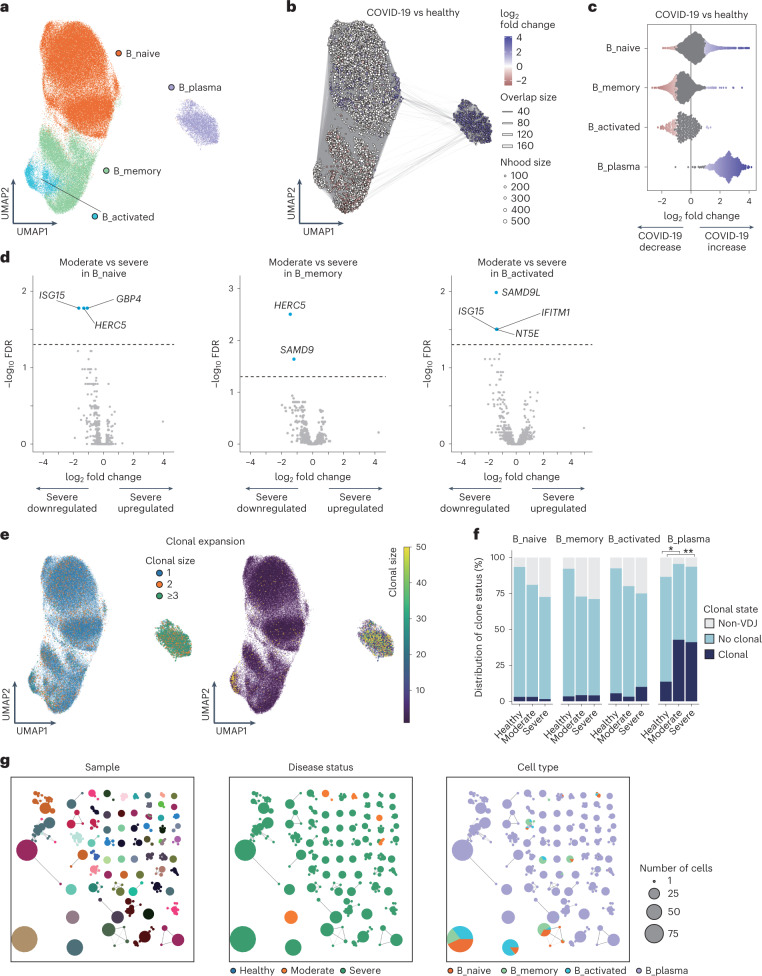
Differential abundance, gene expression and clonal analysis of B cells. **a**, UMAP embedding of four cell types of 123,728 B cells (Extended Data Fig. 4a). **b**, Graph representation of Nhoods identified by Milo. Nodes are Nhoods, colored by their log_2_ FC between COVID-19 (*n* = 72) and healthy controls (*n* = 75) adjusted by age and sex. Nondifferential abundance Nhoods (FDR ≥ 0.1) are colored white. **c**, Beeswarm plot showing the distribution of adjusted log_2_ FC in abundance between COVID-19 and healthy controls in Nhoods according to four cell types. **d**, The differential gene expression analysis between moderate (*n* = 8) and severe (*n* = 64) COVID-19 in B_naive, B_memory and B_activated. DEGs (FDR < 0.05 and FC > 2) are colored in light blue and labeled by gene symbols. **e**, UMAP embedding of B cells colored by clonal expansion size. Left panel shows clonal expansion divided into three categories, and right panel shows clonal expansion size from 0 to 50. **f**, The distribution of the clone state of B cells in each cluster according to disease status. Differences of average clonal expansion rate of each sample between disease status were evaluated in each cluster using two-sided Welch’s *t*-test (*P_uncorrected_ = 0.012, **P_uncorrected_ = 1.3 × 10^−7^), where only cells mapped with BCRs were included in the analysis (*n* = 75 healthy, *n* = 9 moderate, *n* = 64 severe). **g**, Network plots showing similarity of CDR3 amino acid sequence in BCR heavy and light chain for each sample, disease status and cell types. Clonotype clusters with clonal size ≥10 are selected.

**Extended Data Fig. 1:**
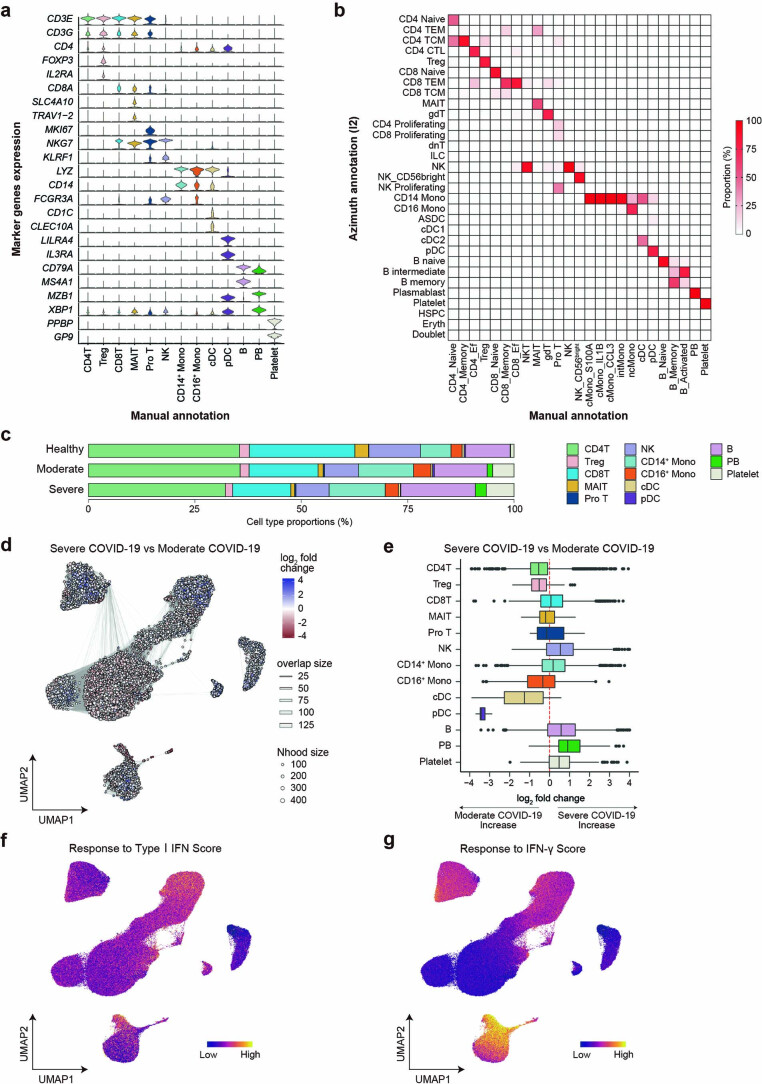
Cell type annotation of PBMC, differential abundance analysis and IFN responses. (**a**) Violin plots showing the expression distribution of selected canonical cell markers in the 13 clusters. The rows represent selected marker genes and the columns represent clusters. (**b**) Tile plot showing percentage concordance between the manually annotated 25 clusters and Azimuth annotation. (**c**) A bar plot of the proportion of cell types shown in Fig. 1b, separated by three conditions (*n* = 75 healthy, *n* = 9 moderate, *n* = 64 severe). (**d**) Graph representation of neighborhoods identified by Milo in COVID-19 patients. Nodes are neighborhoods, colored by their log_2_ fold change between moderate (*n* = 8) and severe (*n* = 64) COVID-19 patients adjusted by age, sex, time since symptom onset and duration of systemic steroids treatment. Nhood, neighborhood. (**e**) Box plot showing the distribution of adjusted log_2_ fold change in abundance between moderate and severe COVID-19 in neighborhoods according to 13 cell types. Boxes denote the interquartile range (IQR), and the median is shown as horizontal bars. Whiskers extend to 1.5 times the IQR, and outliers are shown as individual points. (**f,g**) UMAP embedding of PBMCs colored by Type I IFN response score and IFN-γ response score. The score was calculated using a gene set termed ‘GOBP_RESPONSE_TO_TYPE_I_INTERFERON’ (GO:0034340) and ‘GOBP_RESPONSE_TO_INTERFERON_GAMMA’ (GO:0034341), respectively.

**Extended Data Fig. 2:**
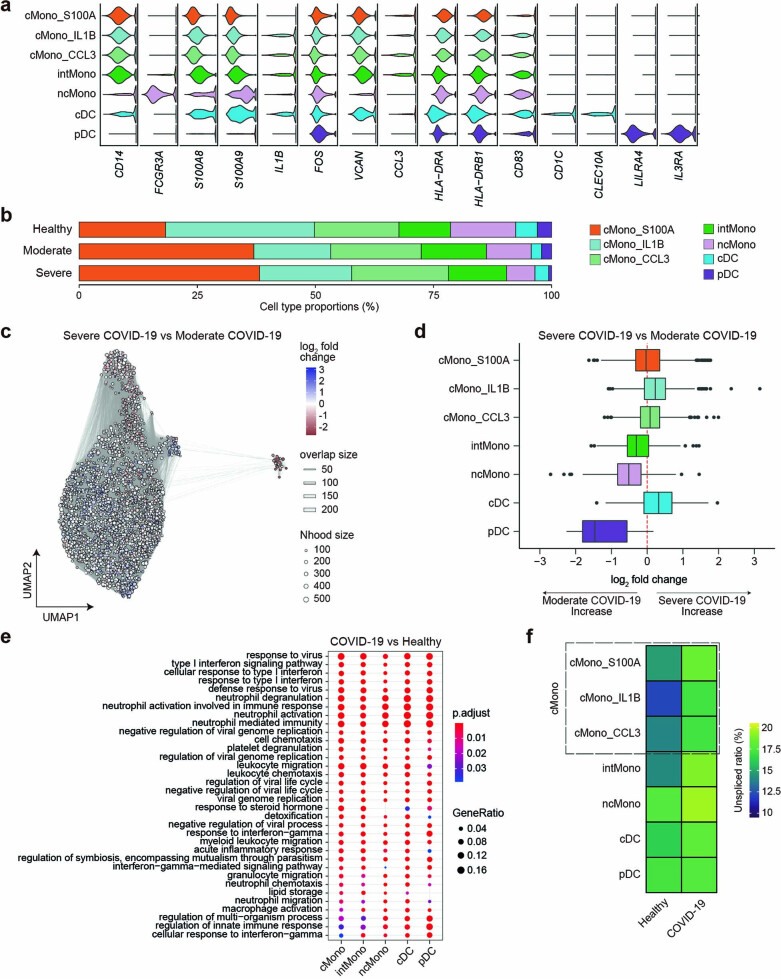
Immunological features of monocytes and dendritic cells. (**a**) Violin plots showing the expression distribution of selected canonical cell markers in the seven clusters. The rows represent clusters and the columns represent selected marker genes. (**b**) A bar plot of the proportion of cell types, separated by three conditions (*n* = 75 healthy, *n* = 9 moderate, *n* = 64 severe). (**c**) Graph representation of neighborhoods identified by Milo in COVID-19 patients. Nodes are neighborhoods, colored by their log_2_ fold change between moderate (*n* = 8) and severe (*n* = 64) COVID-19 patients adjusted by age, sex, time since symptom onset and duration of systemic steroids treatment. Nhood, neighborhood. (**d**) Box plot showing the distribution of adjusted log_2_ fold change in abundance between moderate and severe COVID-19 in neighborhoods according to seven cell types. Boxes denote the interquartile range (IQR), and the median is shown as horizontal bars. Whiskers extend to 1.5 times the IQR, and outliers are shown as individual points. (**e**) The top 20 enriched biological processes by GO analysis of upregulated DEGs of COVID-19 (*n* = 72) compared to healthy controls (*n* = 75) in five cell types. Dot color indicates the statistical significance of the enrichment (adjusted *P*-values via the Benjamini-Hochberg method), and dot size represents gene ratio annotated to each term. (**f**) Heatmaps depicting the average unspliced ratio of seven cell types stratified by COVID-19 (*n* = 73) and healthy controls (*n* = 75).

**Extended Data Fig. 3:**
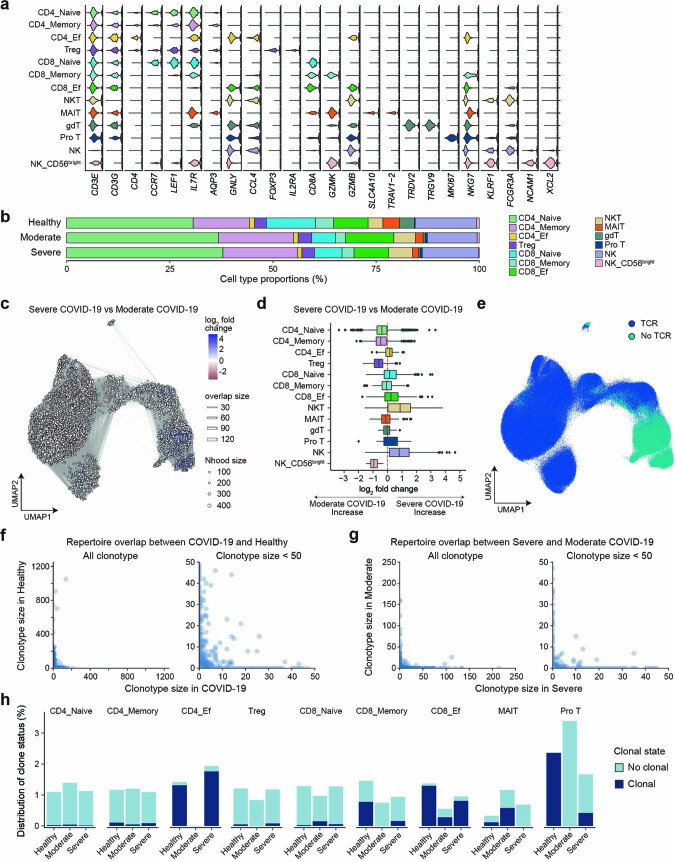
Immunological features of T cells and NK cells and TCR analysis. (**a**) Violin plots showing the expression distribution of selected canonical cell markers in 13 clusters. The rows represent clusters and the columns represent selected marker genes. (**b**) A bar plot of the proportion of cell types, separated by three conditions (*n* = 75 healthy, *n* = 9 moderate, *n* = 64 severe). (**c**) Graph representation of neighborhoods identified by Milo in COVID-19 patients. Nodes are neighborhoods, colored by their log_2_ fold change between moderate (*n* = 8) and severe (*n* = 64) COVID-19 patients adjusted by age, sex, time since symptom onset and duration of systemic steroids treatment. Nhood, neighborhood. (**d**) Box plot showing the distribution of adjusted log_2_ fold change in abundance between moderate and severe COVID-19 in neighborhoods according to 13 cell types. Boxes denote the interquartile range (IQR), and the median is shown as horizontal bars. Whiskers extend to 1.5 times the IQR, and outliers are shown as individual points. (**e**) UMAP embedding of TCR detection. (**f**) Repertoire overlap according to clonotype size between COVID-19 (*n* = 73) and healthy controls (*n* = 75) in all clonotypes (left) and in clonotypes with size < 50 (right). (**g**) Repertoire overlap according to clonotype size between moderate (*n* = 9) and severe (*n* = 64) group in all clonotypes (left) and in clonotypes with size < 50 (right). (**h**) The distribution of the clonal state of T cells which were suspected to be specific to SARS-CoV-2 based on CDR3 amino acid sequence in each cluster according to disease status (*n* = 75 healthy, *n* = 9 moderate, *n* = 64 severe).

**Extended Data Fig. 4:**
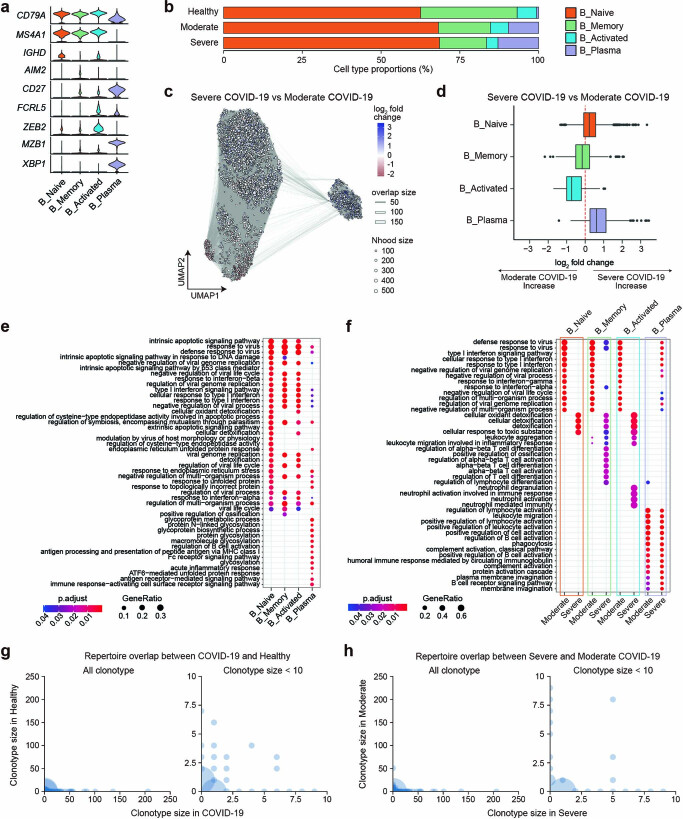
Immunological features of B cells and BCR analysis. (**a**) Violin plots showing the expression distribution of selected canonical cell markers in four clusters. (**b**) A bar plot of the proportion of cell types, separated by three conditions (*n* = 75 healthy, *n* = 9 moderate, *n* = 64 severe). (**c**) Graph representation of neighborhoods identified by Milo in COVID-19 patients. Nodes are neighborhoods, colored by their log_2_ fold change between moderate (*n* = 8) and severe (*n* = 64) COVID-19 patients adjusted by age, sex, time since symptom onset and duration of systemic steroids treatment. Nhood, neighborhood. (**d**) Box plot showing the distribution of adjusted log_2_ fold change in abundance between moderate and severe COVID-19 in neighborhoods according to four cell types. Boxes denote the interquartile range (IQR), and the median is shown as horizontal bars. Whiskers extend to 1.5 times the IQR, and outliers are shown as individual points. (**e**) The top 20 enriched biological processes by GO analysis of upregulated DEGs of COVID-19 (*n* = 72) compared to healthy controls (*n* = 75) in four cell types. Dot color indicates the statistical significance of the enrichment (adjusted *P*-values via the Benjamini-Hochberg method), and dot size represents gene ratio annotated to each term. (**f**) The top 10 enriched biological processes by GO analysis of upregulated DEGs of moderate (*n* = 8) and severe (*n* = 64) compared to healthy group (*n* = 75). Dot color and dot size mean the same as in (**e**). (**g**) Repertoire overlap according to clonotype size between COVID-19 (*n* = 73) and healthy controls (*n* = 75) in all clonotypes (left) and in clonotypes with size < 10 (right). (**h**) Repertoire overlap according to clonotype size between moderate (*n* = 9) and severe (*n* = 64) group in all clonotypes (left) and in clonotypes with size < 10 (right).

After the unified single-cell analysis pipeline (Methods), we obtained 895,460 high-quality cells from PBMCs of all the samples. Cells were manually annotated based on the RNA expression of known marker genes to discriminate subpopulations^6,7,24,25^. We defined 13 cell subsets (Fig. 1b and Extended Data Fig. 1a) and further identified 25 cell states by following subclustering (Figs. 2a, 3a and 4a, Extended Data Figs. 2a, 3a and 4a and Supplementary Table 2). Cell annotation was validated using Azimuth^26^ (Extended Data Fig. 1b).

To reveal the compositional changes between COVID-19 and healthy controls, we applied Milo^27^, identifying 39,170 neighborhoods, of which 21,279 showed evidence of differential abundance (FDR < 0.1, Fig. 1c). We found a prominent decrease in T cells, NK cells and DC for COVID-19 patients compared to healthy controls, consistent with the previous reports^13,28^ (Fig. 1d and Extended Data Fig. 1c). Next, we evaluated differential abundance with disease severity, identifying 14,350 neighborhoods, of which 76 showed significant differential abundance (Extended Data Fig. 1d). While few neighborhoods showed significant differential abundance, there was a trend toward increased B cells and plasmablasts (PB) and decreased DC in severe COVID-19 (Extended Data Fig. 1e).

The dysregulated IFN response has been suggested in COVID-19 (refs. ^8,14,15,29^), leading us to evaluate IFN response across PBMCs. We first defined a score of response to Type I IFN and that of response to IFN-γ for each cell based on the expression of a published gene list. We observed high expressions of Type I IFN response genes in NK and CD16^+^ monocytes and higher response for moderate COVID-19 compared to the other conditions, especially in monocytes and DC (Fig. 1e, Extended Data Fig. 1f and Supplementary Table 3). We also found high expressions of IFN-γ response genes in CD16^+^ monocytes and conventional dendritic cells (cDC), and higher response for moderate COVID-19, especially in CD16^+^ monocytes and pDC (Fig. 1f, Extended Data Fig. 1g and Supplementary Table 3). These data are consistent with the previous reports that systemic IFN response is higher for nonsevere disease^14,29^, suggesting the potential importance of innate immune cells in immunopathology of COVID-19.

### Monocytes and DC in COVID-19

To characterize transcriptome dynamics in 116,944 cells annotated as monocytes and DC across disease conditions, we subclustered monocytes into five subsets and DC into two subsets according to the expression of canonical gene markers as follows: cMono_S100A, cMono_IL1B, cMono_CCL3, intermediate monocytes (intMono), ncMono, cDC and pDC (Fig. 2a and Extended Data Fig. 2a).

To reveal the compositional changes between COVID-19 and healthy controls in this subset, we performed differential abundance analysis using Milo^27^, identifying 6,721 neighborhoods, of which 1,265 showed evidence of differential abundance (Fig. 2b). The proportion of ncMono, cDC and pDC declined prominently in COVID-19 patients compared to healthy controls (Fig. 2c and Extended Data Fig. 2b). Next, we evaluated differential abundance with disease severity, identifying 3,003 neighborhoods, of which 19 showed significant differential abundance (Extended Data Fig. 2c). While few neighborhoods showed significant differential abundance, there was a trend toward a decrease in pDC and ncMono in severe compared to moderate COVID-19 (Extended Data Fig. 2d), implying that ncMono might contribute to immunopathology of COVID-19 severity because decreased cell proportion of ncMono is one of the COVID-19-specific features^13,30,31^.

To gain insight into functions of different cell subsets according to COVID-19 severity, we performed differential expression analyses and a set of Gene Ontology (GO) analyses across the groups (that is, all COVID-19 patients versus healthy, moderate disease versus healthy and severe disease versus healthy) in the five subsets (cMono, intMono, ncMono, cDC and pDC). The top 20 enriched pathways upregulated in COVID-19 versus healthy group were related to innate immunity or antiviral response, and almost all were shared among the five subsets (Extended Data Fig. 2e). We next compared the enriched pathways upregulated in moderate severity versus healthy group and those in severe versus healthy group. The top ten enriched pathways were also almost the same in each subset, while there existed several pathways with different enrichment patterns between the moderate disease and severe group (Fig. 2d). Although ‘response to IFN-γ’ pathway was enriched in moderate COVID-19 of each subset, it was not evident in three monocyte subsets in severe COVID-19, suggesting that decreased IFN-γ response in monocyte subsets might contribute to the severity of COVID-19. Enrichment of ‘response to Type I IFN’ pathway was specifically depleted in ncMono of severe COVID-19.

To further elucidate the mechanism of COVID-19 severity, we conducted differential expression analysis between moderate disease versus severe disease group in ncMono. *PLD4*, which digests ssRNA and ssDNA^32^, was the most downregulated in severe compared to moderate disease group (Fig. 2e). In addition, the expression of *CXCL10*, which belongs to IFN-γ-induced gene and critical in response to various infectious pathogens^33^ and has been reported to be involved in COVID-19 severity with proteomics analysis^5,13^, was also prominently decreased in severe group.

To analyze how the dynamics of transcriptional activation and cell transition differ in disease status, we performed RNA velocity analysis^34^. The transition potential from intMono to ncMono was observed in healthy controls, while such transition was not observed in COVID-19 (Fig. 2f). To quantitatively compare the differences of estimated cell transition from cMono to ncMono through intMono^35^ between COVID-19 and healthy controls, we analyzed the increase of unspliced fractions from cMono to ncMono. We found that condition-specific regression slope was lower in COVID-19 patients with significant interaction between three monocytes and two clinical conditions (*P*_Interaction_ = 2.0 × 10^−4^; Fig. 2g and Extended Data Fig. 2f). These data suggest that the decreased proportion of ncMono in COVID-19 patients is a consequence of the downregulation of the cellular transition from cMono to ncMono.

### T cells and NK cells and T cell repertoires in COVID-19

We subclustered 628,715 cells manually annotated as T cells and NK cells from PBMCs and obtained 13 subsets according to RNA expression of canonical markers (Fig. 3a and Extended Data Fig. 3a).

To reveal the compositional changes between COVID-19 and healthy controls, we performed differential abundance analysis using Milo^27^, identifying 27,182 neighborhoods, of which 10,035 showed evidence of differential abundance (Fig. 3b). The proportion of almost all clusters declined in COVID-19 patients (Fig. 3c and Extended Data Fig. 3b). We next evaluated differential abundance with disease severity, identifying 7,981 neighborhoods, of which nine showed significant differential abundance (Extended Data Fig. 3c). Although there were few neighborhoods showing significant differential abundance, the proportions of CD4T cells, regulatory T (T_reg_) cells and CD56^bright^ NK (NK_CD56^bright^) cells tended to be lower in severe COVID-19 compared to moderate COVID-19, while those of natural killer T (NKT) cells and NK tended to be higher (Extended Data Fig. 3d).

To gain insight into the clonal relationship among individual T cells across three disease conditions, we performed T cell receptor (TCR) analysis for nine subsets except for γδ T (gdT) cells, NKT, NK and NK_CD56^bright^. The detection percentage of TCRs was 73.1% (Fig. 3d and Extended Data Fig. 3e). Similar to the previous report^6,36^, the large clonal expansions were observed particularly in CD4^+^ effector T (CD4_Ef) cells, CD8^+^ effector T (CD8_Ef) cells and mucosal-associated invariant T (MAIT) cells (Fig. 3e,f). The proportion of clonally expanded CD4_Ef increased in moderate disease compared to healthy and severe group (Fig. 3d and Supplementary Table 4), suggesting that efficient clonal expansion of CD4_Ef might contribute to the prevention of serious COVID-19. We next examined whether expanded clonotypes were shared among each sample, between COVID-19 and healthy controls, and among cell types. The large expanded clonotypes exhibited parallel expansion in several cell types, particularly in CD4_Ef, CD8^+^ memory T (CD8_memory) cells and CD8_Ef, whereas a vast majority of these expanded clonotypes were unique for individual patients (Fig. 3f and Extended Data Fig. 3f,g). In contrast, the large expanded clonotypes in MAIT were shared among individuals.

We compared each clonotype from our data against the currently known CDR3 sequences of SARS-CoV-2-specific TCR in VDJdb^37^. A small number of our TCRs (*n* = 4,143; 0.8%) shared their CDR3 with reported SARS-CoV-2-specific TCRs (Fig. 3g). The distribution of TCR specific to SARS-CoV-2 was relatively uniform across cell types, and the clonal expansions of such TCRs were observed in memory or activated T cells (Fig. 3g and Extended Data Fig. 3h). Considering the low percentage of SARS-CoV-2-specific TCRs and the low sharing of expanded TCRs among individuals, further accumulation of data on SARS-CoV-2-specific TCRs would be warranted.

### B cells and B cell repertoires in COVID-19

We subclustered 123,728 cells manually annotated as B cells and PB from PBMCs and obtained four subsets according to RNA expression of canonical markers (Fig. 4a and Extended Data Fig. 4a).

To reveal the compositional changes between COVID-19 and healthy controls, we performed differential abundance analysis using Milo^27^, identifying 8,169 neighborhoods, of which 2,120 showed evidence of differential abundance (Fig. 4b). The proportion of B_naive and B_plasma increased in COVID-19 patients, whereas that of B_memory and B_activated decreased (Fig. 4c and Extended Data Fig. 4b). We also evaluated differential abundance with disease severity, identifying 4,006 neighborhoods, of which none showed significant differential abundance. However, there was a trend toward a higher proportion of B_plasma in severe compared to the moderate disease group, while that of B_activated tended to be lower (Extended Data Fig. 4c,d).

We next performed differential expression and pathway enrichment analysis in four subsets. Pathway enrichment analysis showed that pathways related to antiviral response and immune response were enriched in COVID-19 patients compared to healthy controls (Extended Data Fig. 4e), and that the biological pathways related to IFN response were enriched in three subsets (B_naive, B_memory and B_activated) in the moderate disease group, while those pathways were not in severe group (Extended Data Fig. 4f), consistent with a previous report^5^. Conversely, pathways related to Type I IFN were enriched in B_plasma in severe group, but not in the moderate severity group (Extended Data Fig. 4f). We also conducted DE analysis between moderate versus severe COVID-19. *HERC5*, which has direct antiviral function by catalyzing ISGylation^38^, was significantly downregulated in B_naive and B_memory of severe compared to moderate COVID-19 (Fig. 4d). IFN-related genes, such as *ISG15*, *IFITM1* and *GBP4*, were also downregulated in B_naive and B_activated of severe compared to moderate COVID-19 (Fig. 4d).

Finally, we performed B cell receptor (BCR) analysis. The detection percentage of BCRs was more than 80% in B cell subset (Fig. 4e,f). Clonal expansions were most evident in B_plasma, which showed the larger expansion in COVID-19 (Fig. 4e,f and Supplementary Table 5). We next examined whether expanded clonotypes were shared among each sample, between COVID-19 and healthy controls, and among cell types (Fig. 4g and Extended Data Fig. 4g,h). In contrast to TCR analysis, very few clonotypes were shared between COVID-19 and healthy controls (Fig. 4g and Extended Data Fig. 4g). In addition, while the expanded clonotypes in B_plasma were not shared with the other B cell subsets, the expanded clonotypes exhibited parallel expansion among B_naive, B_memory and B_activated (Fig. 4g). Together, our results suggest that in COVID-19 disease, a robust antibody response characterized by clonally expanded circulating PB occurs against a background of augmented IFN responses.

### Changes of intercellular interactions in PBMC across clinical status

**Fig. 5:**
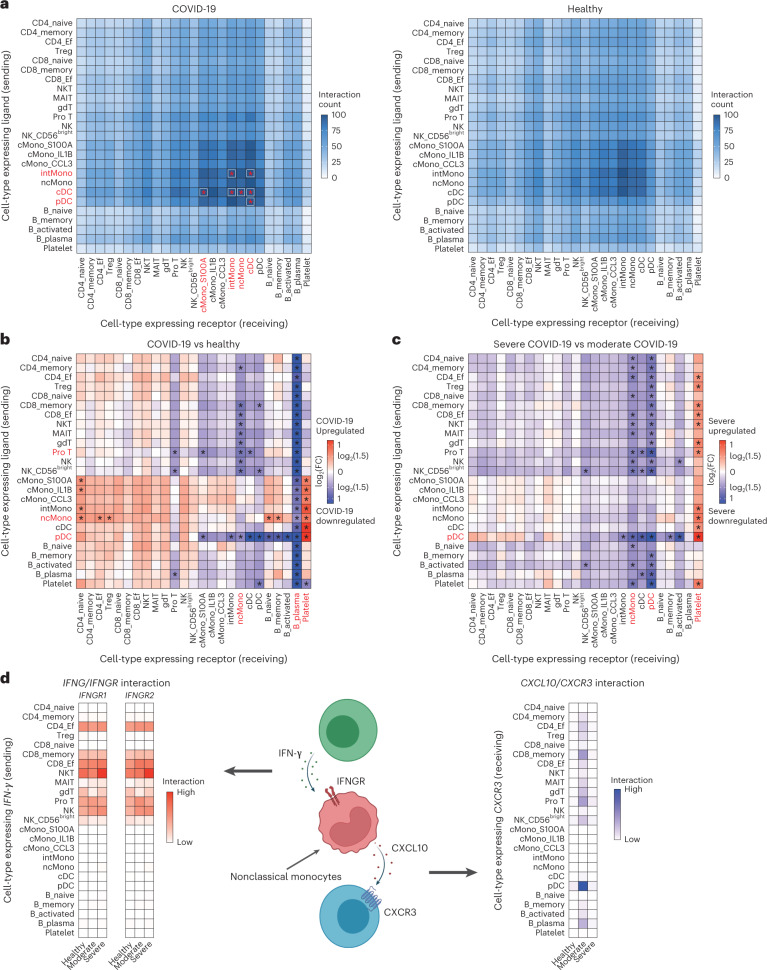
Differential cell–cell interactions between COVID-19 patients and healthy controls and within COVID-19 severity. **a**, Heatmaps depicting number of ligand–receptor pairs connecting cell–cell pairs for COVID-19 (*n* = 73) and healthy controls (*n* = 75), respectively. Rows indicate cells expressing the ligands and columns indicate cells expressing the receptors. An asterisk indicates cell–cell interactions with a number of ligand–receptor of more than 100, and cell types that share such interactions with at least one cell type are highlighted in red. **b**, Heatmap depicting the cell-connectivity-summary networks based on mean expression weight between COVID-19 (*n* = 73) and healthy controls (*n* = 75). An asterisk indicates the cell–cell interactions with FC of mean expression ≥1.5, and cell types that share cell–cell interactions with FC of mean expression ≥1.5 with five or more cell types are highlighted in red. **c**, Heatmap depicting the cell-connectivity-summary networks based on mean expression weight between moderate (*n* = 9) and severe (*n* = 64) COVID-19. An asterisk and red highlight mean the same as in **b**. **d**, Cell–cell interaction of *IFNG/IFNGR* and *CXCL10/CXCR3* around nonclassical monocytes. Heatmaps depicting the cell-connectivity-summary networks based on mean expression weight of *IFNG/IFNGR* (left) and *CXCL10/CXCR3* (right) according to three conditions, respectively (*n* = 75 healthy, *n* = 9 moderate, *n* = 64 severe). The image was created using BioRender.com. Ef, effector.

**Extended Data Fig. 5:**
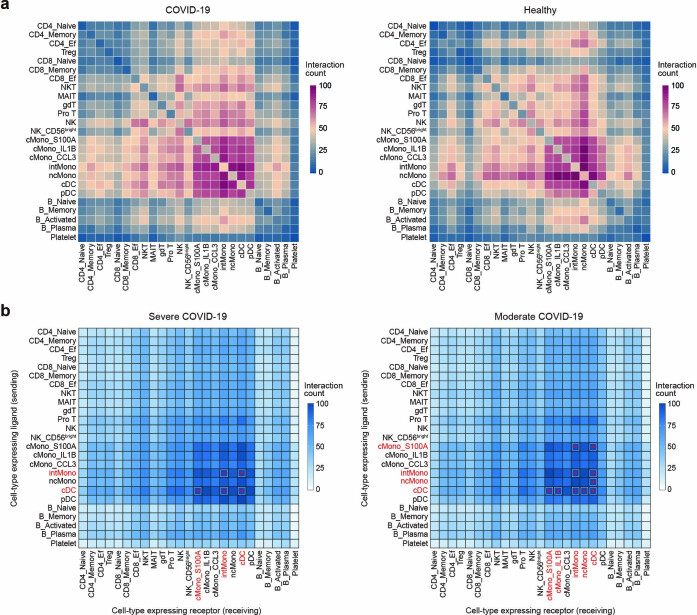
Cell-cell interaction analysis using CellPhoneDB and NATMI. (**a**) Heatmaps depicting cell-cell communications quantified by CellPhoneDB in COVID-19 (*n* = 73) and healthy controls (*n* = 75), respectively. (**b**) Heatmaps depicting cell-cell communications quantified by NATMI in moderate (*n* = 9) and severe (*n* = 64) group, respectively. Rows indicate cells expressing the ligands and columns indicate cells expressing the receptors. Asterisk indicates cell-cell interactions with a number of ligand-receptor more than 100, and cell types which share more than 100 cell-cell interactions with at least one cell type are highlighted in red.

To map the cellular interaction differences between COVID-19 and healthy controls, we inferred all possible intercellular communications by the expression of ligand–receptor pairs in both cell populations using CellPhoneDB^39^ and NATMI^40^. CellPhoneDB and NATMI revealed strong interactions particularly among monocytes and DC in both COVID-19 and healthy groups (Fig. 5a and Extended Data Fig. 5a).

In addition to simple edge count analysis, we examined the differences in the cell-connectivity-summary networks based on mean expression weight between COVID-19 and healthy controls using NATMI^40^. The cellular interactions involving pDC as the sender in COVID-19 patients were lower than in healthy controls, and those involving B_plasma as a receiver in COVID-19 patients were lower than in healthy controls (Fig. 5b).

We next investigated the differences in the cell-connectivity-summary networks based on mean expression weight between moderate and severe COVID-19. Almost all intercellular interactions were reduced in severe compared to moderate disease group (Fig. 5c and Extended Data Fig. 5b). Notably, the intercellular interactions signaling from pDC and those signaling to ncMono and pDC were reduced in severe COVID-19, implying that the dysfunction of the intercellular interactions involving these two subsets might be related to the severity of COVID-19.

Finally, we explored the intercellular interactions centered on *CXCL10* in ncMono, which was significantly downregulated in severe COVID-19 (Fig. 2e). *CXCL10* is an IFN-γ-induced gene and exerts its biological effects by binding to *CXCR3* (ref. ^33^). Therefore, we investigated *IFNG*/*IFNGR* interactions of ncMono as receiver and *CXCL10/CXCR3* interaction of ncMono as sender using NATMI (Fig. 5d). Activated T cells and NK cells showed the strong interactions of *IFNG/IFNGR* with ncMono, which were enhanced as the severity of COVID-19 increased. On the other hand, pDC and to a lesser extent activated T cells showed the *CXCL10*/*CXCR3* interaction with ncMono in moderate COVID-19, which was not observed in healthy controls and severe COVID-19 (Fig. 5d). These intercellular interaction analyses computationally infer the possibility that dysfunction of ncMono and the consequently reduced interaction of *CXCL10*/*CXCR3* might be one of the factors responsible for driving COVID-19 severity.

### Host genetics risks of the severity of COVID-19 in PBMC

Elucidating interaction between host genetics and transcriptional dynamics resolves causal biological mechanism of infection. To evaluate genome-wide host genetics risk of COVID-19 and identify subpopulations of disease-associated cells in PBMCs, we integrated information from our scRNA-seq data with polygenic signals from COVID-19 GWAS using scDRS^41^.

**Fig. 6:**
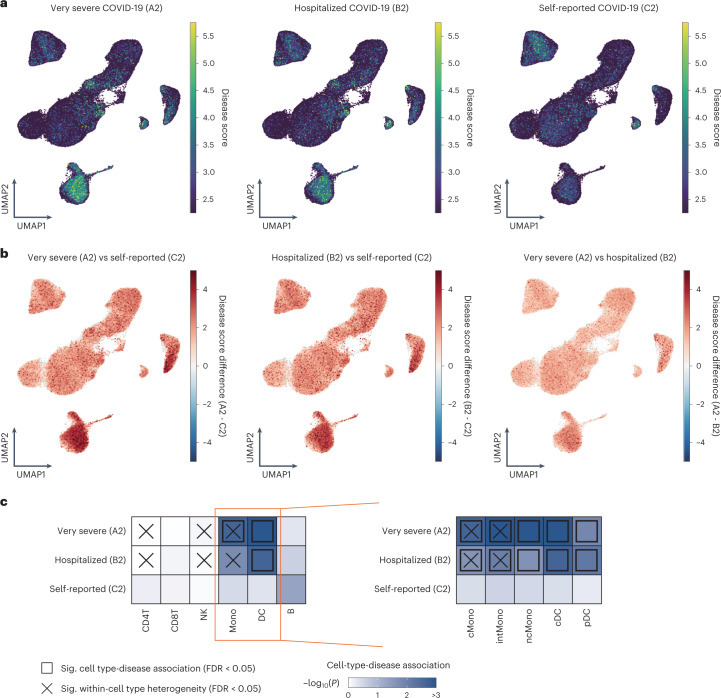
Associations of PBMC cell types with host genetic risk of COVID-19. **a**, UMAP embedding of COVID-19 PBMCs (*n* = 72) colored by scDRS disease score calculated from GWAS summary statistics of three phenotypes from COVID-19 HGI (round 6). **b**, Differences of disease score in individual cell-level among three phenotypes. **c**, Heatmaps depicting each cell type-disease association for three phenotypes, respectively. Heatmap colors denote uncorrected *P* value of cell-type-disease association evaluated using scDRS. Squares denote significant cell type-disease associations (FDR < 0.05), and cross symbols denote significant heterogeneity in association with disease across individual cells within a given cell type (FDR < 0.05). FDR was calculated via the Benjamini–Hochberg method across all pairs of cell types and three phenotypes.

**Extended Data Fig. 6:**
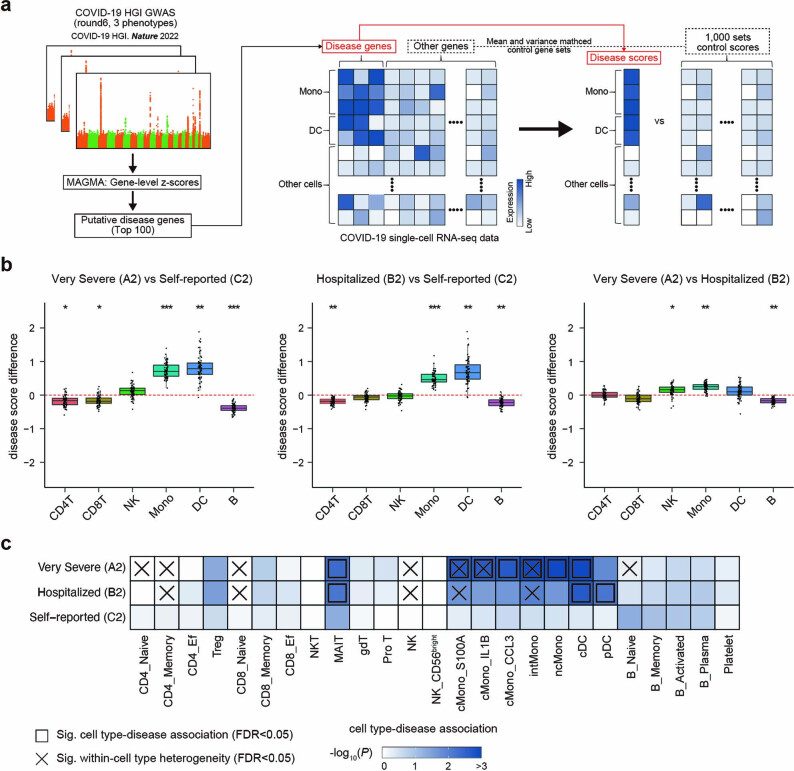
Assessing the association of PBMC cell types with host genetic risk. (**a**) Flowchart of integrated analysis of scRNA-seq data and COVID-19 HGI (round6) GWAS summary statistics using scDRS. The figure design is based on the original paper of scDRS^41^. (**b**) Differences of scDRS disease score between COVID-19 phenotypes in six major cell types. Differences of average disease scores of each sample were evaluated using two-sided paired *t*-test (adjusted for multiple comparisons using Bonferroni’s correction across all pairs of six cell-types and three GWAS comparisons, *P < 1×10^-10^, **P < 1×10^-20^, ***P < 1×10^-30^). Boxes denote the interquartile range (IQR), and the median is shown as horizontal bars. Whiskers extend to 1.5 times the IQR, and all COVID-19 patients (*n*　= 72) are shown as individual points. (**c**) Heatmaps depicting cell type-disease association for three phenotypes in the manually annotated 25 clusters of COVID-19 scRNA-seq dataset. Heatmap colors denote uncorrected *P*-value of cell type-disease association evaluated using scDRS. Squares denote significant cell type-disease associations (FDR < 0.05), and cross symbols denote significant heterogeneity in association with disease across individual cells within a given cell type (FDR < 0.05). FDR was calculated via the Benjamini-Hochberg method across all pairs of 25 cell types and three GWAS phenotypes.

First, we computed a disease score for each cell observed at our COVID-19 scRNA-seq datasets according to the COVID-19 case-control GWAS summary statistics of COVID-19 HGI (round 6; ref. ^18^), and projected the scores on Uniform manifold approximation and projection (UMAP; Extended Data Fig. 6a). The disease scores at the COVID-19 scRNA-seq dataset were similar across any cell types when using the GWAS summary statistics of self-reported COVID-19 (C2, *n*_Case_ = 112,612), while the cells annotated as monocytes and DC showed the higher disease scores than the other cell types when GWAS cases were restricted to severe ones (that is, hospitalized COVID-19 (B2, *n*_Case_ = 24,274) and very severe COVID-19 (A2, *n*_Case_ = 8,779); Fig. 6a). When comparing disease score for each phenotype, disease scores from severe GWASs were higher than those from self-reported GWAS, particularly at cells annotated as monocytes and DC, which was more prominent in very severe GWAS (Fig. 6b, Extended Data Fig. 6b and Supplementary Table 6). Next, we assessed associations between the six major cell types (Methods) and three COVID-19 GWAS phenotypes, and also within-cell type association heterogeneity using scDRS^41^. No cell type was enriched in the self-reported infection GWAS, whereas monocytes were associated with very severe GWAS, and DC was associated with hospitalization and very severe GWASs (Fig. 6c and Supplementary Table 7), demonstrating that polygenic risks involved in the severity of COVID-19 were enriched in the cells responsible for innate immunity. In addition, three cell types showed heterogeneity in association with hospitalization and critical illness (Fig. 6c). To investigate the host genetics association of monocytes and DC with severe COVID-19 in more detail, we examined cell type-disease association and its heterogeneity of the five innate immune subsets (cMono, intMono, ncMono, cDC and pDC). All of these subsets were associated with GWAS for severe disease, with stronger associations in very severe cases (Fig. 6c and Supplementary Table 7). Some of them showed significant heterogeneity within the subset (Fig. 6c and Supplementary Table 7). The subset analysis of manually annotated 25 clusters revealed the significant association of MAIT with GWAS for severe disease in addition to monocytes and DC subsets (Extended Data Fig. 6c and Supplementary Table 7). MAIT functions as innate sensors of viral infection^42^, again implicating the involvement of host genetic risk of severe COVID-19 with innate immunity.

### Context and cell-type-specific eQTLs of COVID-19-related variants

**Extended Data Fig. 7:**
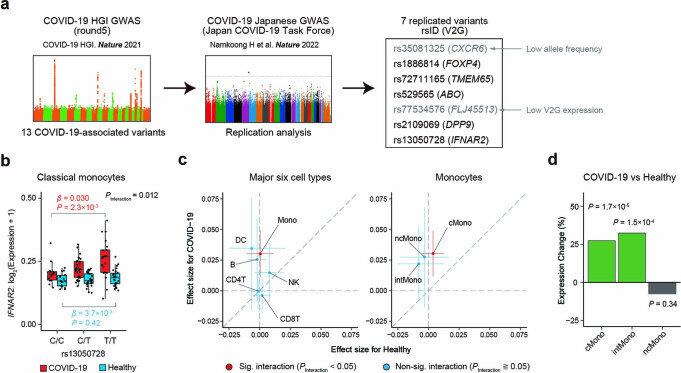
Single-cell eQTL analysis of rs13050728 and differential expression analysis of *IFNAR2*. (**a**) Flowchart of how to select COVID-19-associated variants in single-cell eQTL analysis. (**b**) rs13050728 eQTL for *IFNAR2* in classical monocytes. The box plot shows the eQTL in COVID-19 (red) and healthy controls (blue). Boxes denote the interquartile range (IQR), and the median is shown as horizontal bars. Whiskers extend to 1.5 times the IQR. All samples are shown as individual points (*n* = 67 COVID-19, *n* = 75 healthy). (**c**) Co-plots of the eQTL effect sizes of rs13050728 and 95% confidence intervals between COVID-19 and healthy controls. Dots represent the effect sizes and bars represent the 95 % confidence intervals. Cell types with significant interaction (*P*_Interaction_ < 0.05) in eQTL effect between COVID-19 and healthy controls are colored in red, and the rest are colored in blue. (**d**) Expression changes of *IFNAR2* in differential expression (DE) analysis between COVID-19 (*n* = 72) vs healthy controls (*n* = 75) are indicated for each cell cluster. Cell types with *P* < 0.05 in DE analysis are colored in green, and the rest is colored in gray. *P*-values are uncorrected and reflect two-sided tests in (**b**-**d**).

To gain a better understanding of transcriptional variability and dynamics regulated by the GWAS-identified COVID-19-associated variants, we examined eQTL effects of the replicated variants at the GWAS of COVID-19 in the Japanese population^20^, separately for COVID-19 (*n* = 67) and healthy controls (*n* = 75; Extended Data Fig. 7a).

**Fig. 7:**
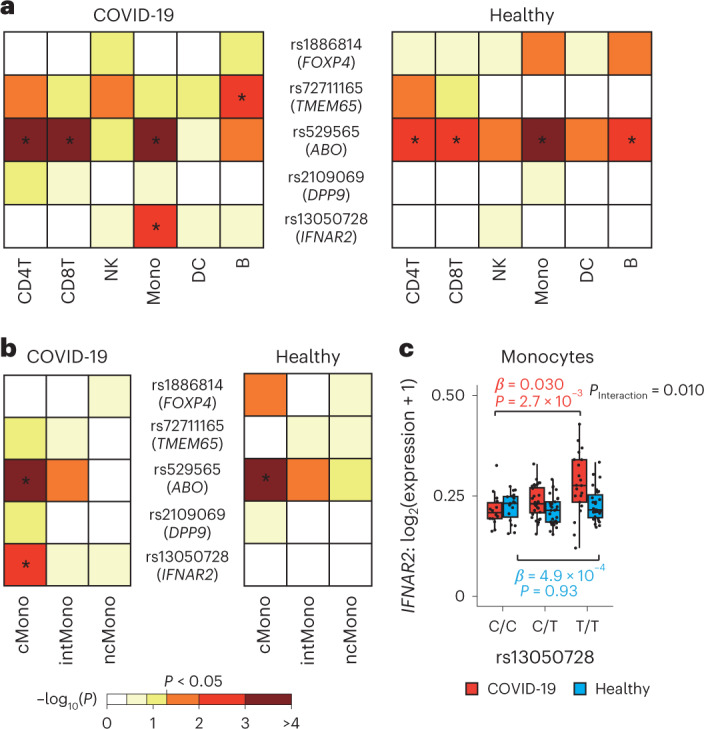
Context-specific and cell type-specific eQTL analysis of COVID-19-associated risk variants. **a**, Heatmaps depicting eQTL effect of COVID-19-associated risk variants on V2G, highest gene prioritized by the V2G score of Open Target Genetics, by six major cell types, separately for COVID-19 (*n* = 67) and healthy controls (*n* = 75). Heatmap colors denote uncorrected *P* value of eQTL effect. **b**, Heatmaps depicting eQTL effect of COVID-19-associated risk variants by three cell types of monocytes, separately for COVID-19 (*n* = 67) and healthy controls (*n* = 75). **c**, rs13050728 eQTL effect on *IFNAR2* expression in monocytes. The box plot shows the eQTL effect in COVID-19 (red) and healthy controls (blue). *P* values are uncorrected and reflect two-sided tests. Boxes denote the IQR, and the median is shown as horizontal bars. Whiskers extend to 1.5 times the IQR. All samples are shown as individual points (*n* = 67 COVID-19, *n* = 75 healthy). In **a** and **b**, an asterisk indicates FDR < 0.05, and FDR was calculated via the Benjamini–Hochberg method across all pairs of the cell types and five variants, separately for COVID-19 and healthy controls. IQR, interquartile range.

First, we performed eQTL analysis for the six major cell types (Methods). COVID-19-associated variants showed different cell type distributions with significant eQTL effects between the COVID-19 patients and healthy controls, demonstrating context-specific eQTL effects (Fig. 7a and Supplementary Table 8). Among them, monocytes of the COVID-19 patients had significant eQTL effects in multiple variants (FDR < 0.02). Given the previous analyses demonstrating the involvement of monocytes with COVID-19 severity in this dataset, we examined eQTL effects in the three subsets of monocytes. The two variants with eQTL effects in monocytes of the COVID-19 patients had significant eQTL effects specifically in cMono (FDR = 2.6 × 10^−6^ for *ABO* and FDR = 0.017 for *IFNAR2*), and no such eQTL effect of the *IFNAR2* variant was observed in the healthy controls (FDR = 0.66; Fig. 7b and Supplementary Table 9).

*IFNAR2* has a key role in Type I IFN signaling pathway^16^, leading us to investigate eQTL effect of the *IFNAR2* variant in more detail. We found COVID-19 context-specific increasing dosage effect of the risk allele (rs13050728-T) on *IFNAR2* expression levels in monocytes (*β* = 0.030, 95% CI = 0.011–0.049, *P* = 2.7 × 10^−3^ for COVID-19 and *β* = 4.9 × 10^−4^, 95% CI = −0.010 to 0.011, *P* = 0.93 for healthy controls), especially in cMono (*β* = 0.030, 95%CI = 0.012–0.049, *P* = 2.3 × 10^−3^ for COVID-19 and *β* = 3.7 × 10^−3^, 95% CI = −5.2 × 10^−3^ to 0.013, *P* = 0.42 for healthy controls; Fig. 7c, Extended Data Fig. 7b and Supplementary Tables 8 and 9). While many cell types showed a larger eQTL effect in COVID-19 than healthy controls, monocytes, in particular cMono, specifically had a significant interaction of eQTL effect between COVID-19 and healthy controls (*P*_Interaction_ = 0.010 for monocytes and *P*_Interaction_ = 0.012 for cMono; Fig. 7c and Extended Data Fig. 7b,c). DE analysis revealed increased *IFNAR2* expression in cMono of COVID-19 patients than healthy controls (*P* = 1.7 × 10^−5^; Extended Data Fig. 7d), consistent with previous findings^5^. Taken together, the risk allele of rs13050728 might contribute to severe COVID-19 by increasing expression of *IFNAR2* in cMono, highlighting the importance of context and cell type-specific eQTL analysis to elucidate host genetical effects in pathophysiology of COVID-19.

## Discussion

Here we reported comprehensive analyses of single-cell transcriptome and TCR and BCR of PBMCs from the COVID-19 patients and healthy controls in Japanese, integrated with host genetics data. Our data presented the dysfunction of monocytes or DC, particularly ncMono, in severe COVID-19, the enrichment of host genetics COVID-19 risks in monocytes or DC, as well as COVID-19 context-specific eQTL effect of the *IFNAR2* variant (rs13050728) in monocytes. These highlighted the biological and host genetic critical involvement of innate immune cells in COVID-19 severity.

While previous studies on IFN and SARS-CoV-2 have focused on Type I IFN due to their robust capacity to interfere with viral replication^43^, several studies have indicated that IFN-γ is also an essential component in the severity of COVID-19 (refs. ^29,44,45^). We found the depleted enrichment of IFN-γ response pathways in monocyte subsets from patients with severe COVID-19, again demonstrating the importance of IFN-γ response for COVID-19. One of the COVID-19-specific features is decreased cell fraction of ncMono in severe disease^13,31^, whereas its fraction generally increases in patients with sepsis and inflammatory disease^46^. This phenomenon was also observed in this study, and we revealed the downregulation of the cellular transitions from cMono to ncMono in COVID-19 patients using RNA velocity analysis. In addition to decreased cell fraction, differential expression analysis revealed the severely downregulated expression of *CXCL10*, which is IFN-γ-induced gene and reported to be involved in COVID-19 severity with proteomics analysis^5,13^, in ncMono for severe COVID-19, and cell–cell communication analysis inferred the possibility that *CXCL10/CXCR3* interaction between ncMono and pDC was depleted whereas ncMono firmly received IFN-γ signal from activated T cells in severe COVID-19. Thus, these findings indicate that ncMono might contribute to immunopathology of COVID-19 severity via decreased cell fraction as well as biological dysfunction. However, the mechanisms of the reduced differentiation to ncMono and the dysfunction of ncMono are still elusive. Multimodal single-cell analysis and in vivo experiments should be warranted in the future.

Human genetic background has been demonstrated to influence the pathogenesis of COVID-19 (refs. ^17–20^). Functional analysis has mostly focused on the variant at *LZTFL1* on 3p21, which showed the strongest severity association in Europeans but conferred a rare frequency of the risk allele in East Asian^47,48^, suggesting the importance of additional studies in non-Europeans. We found that the putative disease genes identified by the GWAS of severe phenotypes showed cell type-specific expressions in monocytes and DC. We also showed that eQTL effects of the COVID-19-associated variants replicated in the Japanese GWAS were context and cell type-specific, with the *IFNAR2* variant, in particular, having COVID-19-specific and monocytes-specific eQTL effect, indicating the host genetic involvement of innate immune cells in COVID-19 severity. Because the context-specific eQTL effects were confirmed, collecting more COVID-19 cases and comparing them with healthy controls will help to clarify the pathogenesis of COVID-19 from the perspective of the host genome.

Collectively, our results motivate us for a detailed examination of ncMono function in the context of COVID-19, as well as to increase sample size to perform integrated analysis with genetic data on a larger scale.

## Methods

### Ethics and specimen collection of PBMC for scRNA-seq

Peripheral blood samples were obtained from patients with COVID-19 (*n* = 73) and healthy controls (*n* = 75) at Osaka University Hospital. Patients with COVID-19 were further categorized into groups of moderate (*n* = 9) and severe (*n* = 64) according to disease severity based on the highest score on the World Health Organization (WHO) Ordinal Scale for Clinical Improvement ever-present (WHO, R&D Blueprint—new coronavirus—COVID-19 Therapeutic Trial Synopsis, 2020; ref. ^23^). Almost all cases were patients who were transferred from nearby general hospitals because of severe or potentially severe illness during treatment and already initiated with systemic corticosteroids therapy at others hospitals according to RECOVERY study^49^. The detailed clinical data are summarized in Supplementary Table 1. Part of the subjects (*n*_COVID-19_ = 30, *n*_Control_ = 31) are described elsewhere^20^. One patient with COVID-19 had the karyotype abnormality and was excluded from the analyses including sex as a covariate. This study strictly follows the principles according to the Declaration of Helsinki, with written informed consent obtained from all participants before sample collection according to regular principles. Ethical approvals were gained from the Ethics Committees of Osaka University. There was no compensation for participants.

### Preparation of single-cell suspensions

For both patients with COVID-19 and healthy controls, blood was collected into heparin tubes and PBMCs were isolated using Leucosep (Greiner Bio-One) density gradient centrifugation according to the manufacturer’s instructions. Blood was processed within 3 h of collection for all samples, and stored at −80 °C until use.

### Droplet-based single-cell sequencing

Single-cell suspensions were processed through the 10X Genomics Chromium Controller following the protocol outlined in the Chromium Single Cell V(D)J Reagent Kits (v1.1 Chemistry) User Guide. Chromium Next GEM Single Cell 5′ Library & Gel Bead Kit v1.1 (PN-1000167), Chromium Next GEM Chip G Single Cell Kit (PN-1000127) and Single Index Kit T Set A (PN-1000213) were applied during the process. Oil droplets of encapsulated single cells and barcoded beads (GEMs) were subsequently reverse-transcribed in a Veriti Thermal Cycler (Thermo Fisher Scientific), resulting in cDNA tagged with a cell barcode and unique molecular index (UMI). Next, cDNA was then amplified to generate single-cell libraries according to the manufacturer’s protocol. Quantification was made with an Agilent Bioanalyzer High-Sensitivity DNA assay (Agilent, High-Sensitivity DNA Kit, 5067-4626). Subsequently amplified cDNA was enzymatically fragmented, end-repaired and polyA tagged. Cleanup/size selection was performed on amplified cDNA using SPRIselect magnetic beads (Beckman-Coulter; SPRIselect, B23317). Next, Illumina sequencing adapters were ligated to the size-selected fragments and cleaned up using SPRIselect magnetic beads. Finally, sample indices were selected and amplified, followed by a double-sided size selection using SPRIselect magnetic beads. Final library quality was assessed using an Agilent Bioanalyzer High-Sensitivity DNA assay. Samples were then sequenced on an Illumina NovaSeq 6000 as paired-end mode.

### Alignment, quantification and quality control of scRNA-seq

Droplet libraries were processed using Cell Ranger 5.0.0 (10X Genomics). Sequencing reads were aligned with STAR (v2.7.2a)^50^ using the GRCh38 human reference genome. Filtered expression matrices generated using Cell Ranger count were used to perform the analysis. We excluded cells that had fewer than the first percentile of UMIs or greater than 99th percentile of UMIs in each sample. We also excluded cells with <200 genes expressed or >10% of reads from mitochondrial genes or hemoglobin genes. Additionally, putative doublets were removed using Scrublet (v0.2.1)^51^ and scds (v1.10.0)^52^ for each sample.

### scRNA-seq computational pipelines and analysis

The R package Seurat (v4.1.0) was used for data scaling, transformation, clustering, dimensionality reduction and most visualization^26^. Data were scaled and transformed using the SCTransform() function, and linear regression was performed to remove unwanted variation due to cell quality (% mitochondrial reads). For integration, we identified 3,000 shared highly variable genes (HVGs) using SelectIntegrationFeatures() function. Principal component analysis (PCA) was run on gene expression, followed by batch correction using harmony (v0.1)^53^. UMAP dimension reduction was generated based on the first 30 harmony-adjusted principal components^54^. A nearest-neighbor graph using the first 30 harmony-adjusted principal components was calculated using FindNeighbors() function, followed by clustering using FindClusters() function.

Cellular identity was determined by finding differentially expressed genes (DEGs) for each cluster using FindMarkers() function with parameter ‘test.use=wilcox’, and comparing those markers to known cell type-specific genes (Extended Data Fig. 1a). At the first round of clustering, we identified 13 cell subsets (Fig. 1b). To identify clusters within each major cell type, we performed a second round of clustering on monocytes/DC (CD14^+^ monocytes, CD16^+^ monocytes, cDC and pDC), T/NK cells (CD4T, T_reg_, CD8T, MAIT, proliferative T cells (Pro_T) and NK) and B cells (B and PB), separately, and then we obtained 25 cell subsets (Extended Data Figs. 1a, 2a, 3a and 4a). The final annotation of PBMCs was compared to the PBMC annotation of Azimuth using Seurat^26^. For each of the 25 clusters in our data, the percentage of cells mapped to each Azimuth annotation was calculated (Extended Data Fig. 1b).

To perform polygenic GWAS signal analysis (Fig. 6c) and single-cell eQTL analysis (Fig. 7a), six major cell types were defined from 25 clusters as follows: CD4_naive, CD4_memory, CD4_Ef and T_reg_ were annotated as CD4^+^ T cells (CD4T); CD8_naive, CD8_memory and CD8_Ef were annotated as CD8^+^ T cells (CD8T); NK and NK_CD56^bright^ were annotated as NK; cMono_S100A, cMono_IL1B, cMono_CCL3, intMono and ncMono were annotated as monocytes (Mono); cDC and pDC were annotated as DC; B_naive, B_memory and B_activated and B_plasma were annotated as B cells (B).

### Differential abundance analysis

We used Milo^27^ (v1.2.0) to test for the differential abundance of cells within defined neighborhoods, between two conditions (that is, COVID-19 versus healthy controls or moderate COVID-19 versus severe COVID-19). We first used the buildGraph function to construct a KNN graph with *k* = 30, using 30 principal components (*d* = 30). Next, we used the make neighborhoods function to assign cells to neighborhoods based on their connectivity over the KNN graph. For computational efficiency, we subsampled 5% for all PBMCs and T cells and 10% for mono/DC and B cells. To test for differential abundance, Milo fit an NB GLM to the counts for each neighborhood, accounting for different numbers of cells across samples using TMM normalization, and use the QL *F*-test with a specified contrast to compute a *P* value for each neighborhood. We included age, sex, days since symptom onset and duration of systemic steroids treatment at the time of specimen collection (the last two variables included only in moderate COVID-19 versus severe COVID-19) as covariates in testNhoods function. To control for multiple testing, we adapted the spatial FDR implemented in Milo and used 10% spatial FDR as a threshold for significance. The spatial FDR and log_2_ foldchange (FC) of number of cells between two conditions in each neighborhood were used for visualization.

### IFN response scoring

To evaluate IFN response across PBMCs, we downloaded a gene set termed ‘GOBP_RESPONSE_TO_TYPE_I_INTERFERON (GO:0034340)’ and ‘GOBP_RESPONSE_TO_INTERFERON_GAMMA (GO:0034341)’ from MSigDB. IFN response scores were evaluated using AddModuleScore() function implemented in Seurat with default parameter. To identify cell types with high IFN response, we calculated the average module scores across each of 13 cell types and compared them with that of all PMBCs. The module scores of cells in each cell type were also compared with the average score of all PBMCs using two-sided one-sample *t*-test. To evaluate IFN response of COVID-19 patients in each cell type, we calculated the average module scores across each of 13 cell types by three clinical statuses and compared the scores of moderate or severe group with those of healthy group. The module scores of cells of moderate or severe group were also compared to those of healthy group in each cell type using two-sided Welch’s *t*-test.

### Differential gene expression analysis and GO enrichment

Differential gene expression analysis was performed among (1) all COVID-19 patients versus healthy controls, (2) moderate patients versus healthy controls, (3) severe patients versus healthy controls and (4) moderate versus severe patients. Donor pseudobulk matrices were first created by aggregating gene counts for each cell type, within each sample. Genes were considered for the analysis if they were expressed (UMI count > 0) in more than 10% of cells per cell type. Samples with more than five cells in a cell type were considered in the analysis of the corresponding cell type. Differential gene expression testing was performed using an NB GLM implemented in the Bioconductor package edgeR (v3.32.0)^55^. We included age, sex, days since symptom onset and duration of systemic steroids treatment at the time of specimen collection (the last two variables included only in moderate COVID-19 versus severe COVID-19) in the model as covariates. Statistically significant DEGs were defined with FDR < 0.05 and FC > 2. To find the function of upregulated DEGs, we used the function compareCluster (fun = “enrichGO,” pvalueCutoff = 0.05, pAdjustMethod = “BH,” OrgDb = “org.Hs.eg.db”, ont = ”BP”) of Clusterprofiler (v3.14.3)^56^.

### Estimation of RNA velocity

Spliced and unspliced transcripts were quantified using dropEst (v0.8.6)^57^. Monocytes and DC clusters were evaluated by RNA velocity analysis using scVelo (v0.2.3)^34^, separately for each of the two conditions. dropEst-derived counts were processed, filtered and normalized before velocity estimation on the basis of the top 2,000 HVGs with at least 20 UMI for both spliced and unspliced transcripts across all cells. The moments facilitated the RNA velocity estimation implemented in function scv.tl.velocity with mode set to ‘dynamical’. The estimated velocities were used to construct a velocity graph representing the transition probabilities among cells by function scv.tl.velocity_graph. Finally, the velocity graph was used to embed the RNA velocities into the UMAP by the function scv.pl.velocity_embedding_stream. The fractions of unspliced counts were adopted to quantitatively compare the differences in estimated cell transition in monocytes between COVID-19 and healthy groups. The average unspliced ratio of each sample for each of the three monocyte clusters was calculated. The difference of increase in unspliced ratio across three monocyte clusters (ordered from cMono < intMono < ncMono) was evaluated using a linear regression model in each of the two conditions. The interaction between three monocyte clusters and two conditions (ordered from healthy < COVID-19) was also evaluated using a linear regression model with cluster, condition and cluster × condition as covariates.

### TCR and BCR analysis

Droplet-based sequencing data for TCR sequences and BCR sequences were aligned and quantified using 5.0.0 (10X Genomics) against the GRCh38 human VDJ reference genome. Filtered annotated contigs for TCR sequences and BCR sequences were analyzed using Scirpy (v0.10.0)^58^. For TCR analysis, we selected T cells that were annotated as following nine cell types via single-cell RNA-seq analysis: CD4_naive, CD4_memory, CD4_Ef, T_reg_, CD8_naive, CD8_memory, CD8_Ef, MAIT and Pro_T (Extended Data Fig. 3a). Only cells with both TCR α-chain (TRA) and TCR β-chain (TRB) remained for the downstream analysis. Each unique TRA(s)–TRB(s) pair was defined as a clonotype. Similarly, for BCR analysis, we selected B cells which were annotated as following four cell types via scRNA-seq analysis: B_naive, B_memory, B_activated and B_plasma (Extended Data Fig. 4a). Only cells with both heavy chain (IGH) and light chain (IGK or IGL) were kept for further analysis. Each unique IGH(s)–IGK/IGL(s) pair was defined as a clonotype.

For TCR and BCR data, clonotypes were defined based on CDR3 amino acid sequences with receptor_arms = ‘any’, metric = ‘alignment’ and default cutoff of ten. If one clonotype was present in at least two cells, cells harboring this clonotype were considered to be clonal and the number of cells with such pairs indicated the degree of clonality of the clonotype. Using barcode information, T cells with prevalent TCR clonotypes and B cells with prevalent BCR clonotypes were projected on UMAP embedding. To evaluate differences of clonal state between disease status in each cluster, the average clonal expansion rate of each sample was evaluated using two-sided Welch’s *t*-test, where only cells mapped with TCRs/BCRs were included in the analysis.

We downloaded VDJdb^37^, a curated database of TCR sequences with known antigen specificities, and then investigated the TCR that was specific to SARS-CoV-2, based on CDR3 amino acid sequences with the same parameter as above.

### Cell–cell interaction analysis in PBMC

At first, to reduce the influence of individual samples contributing a larger number of cells and to speed up computation, we capped the number of cells per sample at 2,500 randomly sampled cells. This was done using the SubsetData() function in Seurat. Genes were adopted if they were expressed in more than 1% of all PBMCs. Putative cell–cell interactions of COVID-19 and healthy were quantified using CellPhoneDB (v2.0.0) and NATMI with default settings^39,40^. To investigate the differences in cell–cell interactions between COVID-19 versus healthy and between moderate versus severe COVID-19, we evaluated FC of mean expression weight using DiffEdges.py implemented in NATMI with default settings. As *CXCL10*/*CXCR3* interaction is not listed on connectomeDB2020, we manually added *CXCL10* as ligand and *CXCR3* as receptor. Then, cell–cell interactions of *CXCL10/CXCR3* and *IFNG/IFNGR* were evaluated for each of the three conditions by mean expression weight using NATMI.

### Polygenic GWAS signals on PBMC

We used MAGMA (v1.07)^59^ to compute gene-level association *P* values and *z*-score from GWAS summary statistics of COVID-HGI (round 6)^18^. We used a reference panel based on individuals of European ancestry in the 1000 Genomes Project and used a 10-kb window around the gene body to map SNPs to genes. We selected the top 100 genes based on MAGMA *P* values as putative disease genes.

We used scDRS (v1.0.1)^41^ to quantify the aggregate expression of putative disease genes derived from GWAS summary statistics using MAGMA (each putative disease gene is weighted by its GWAS MAGMA *z*-score and inversely weighted by its gene-specific technical noise level in the single-cell data) in each cell of COVID-19 scRNA-seq data to generate cell-specific raw disease scores. A 1,000 sets of cell-specific raw control scores were calculated from matched control gene sets (matching the gene set size, mean expression and expression variance of the putative disease genes). Then, we normalized the raw disease score and raw control scores for each cell, producing the normalized disease score and normalized control scores. To compute the scores described above, we used the function scdrs compute-score (--n_ctrl = 1000, --cov-file = age, sex, number of genes per cell and disease severity).

For downstream analysis, we performed cell type-level analyses to associate predefined cell types to disease and assess heterogeneity in association to disease across cells within a predefined cell type using the function scdrs perform-downstream with default settings. To correct multiple testing, FDR was calculated via the Benjamini–Hochberg method across all pairs of cell types and three GWAS phenotypes. To compare scDRS disease scores between COVID-19 phenotypes in six major cell types, the differences in average disease scores of each sample were evaluated using two-sided paired *t*-test (adjusted for multiple comparisons using Bonferroni’s correction).

### Genotype data, quality control and genotype imputation

We performed GWAS genotyping of COVID-19 cases and healthy controls using Infinium Asian Screening Array (Illumina) through collaboration with Japan COVID-19 Task Force (https://www.covid19-taskforce.jp/en/home/). We applied stringent quality control filters to the samples (sample call rate < 0.98, related samples with PI_HAT > 0.175 or outlier samples from East Asian clusters in PCA with HapMap project samples), and variants (variant call rate < 0.99, deviation from Hardy–Weinberg equilibrium with *P* < 1.0 × 10^−6^, or minor allele count < 5). We used SHAPEIT4 software (version 4.2.1)^60^ for haplotype phasing of autosomal genotype data. After phasing, we used Minimac4 software (version 1.0.1)^61^ for genome-wide genotype imputation. We used the population-specific imputation reference panel of Japanese (*n* = 1,037) combined with 1,000 Genomes Project Phase3v5 samples (*n* = 2,504)^62^.

### Single-cell eQTL analysis

We applied pseudobulk approach for single-cell eQTL analysis. First, we performed single-cell-level normalization using scran (v1.18.5)^63^. Gene expression per cell type per sample was then calculated as the mean of log2-transformed counts-per-cell-normalized expression across cells. Samples with more than five cells in a cell type were considered in the analysis of the corresponding cell type. We examined eQTL effects of the replicated variants in Japanese COVID-19 GWAS on V2G, the highest gene prioritized by the V2G score of Open Target Genetic, separately for COVID-19 and healthy controls^20,64^. rs35081325 and rs77534576 were excluded from eQTL analysis due to low allele frequency and low expression of V2G (*FLJ45513*), respectively. For PCA, genes were adopted if they were expressed in more than 1% of all PBMCs.

In the eQTL analysis of COVID-19-associated variants, dosage effects of the variants on the gene expression mean were evaluated using linear regression models with the top two PCs of the genotype data, the top two PCs of the gene expression, age, sex, days since symptom onset, duration of systemic steroids treatment at the time of specimen collection and disease severity (the last three variables included only in COVID-19 analysis) as covariates. In the interaction eQTL analysis of the *IFNAR2* variant (rs13050728), the top two PCs of the genotype data, the top two PCs of the gene expression, age and sex were included as covariates. *R* statistical software (version 4.0.2) was used for the analysis. To correct multiple testing, FDR was calculated via the Benjamini–Hochberg method across all pairs of cell types and five variants, separately for COVID-19 and healthy controls.

### Statistics and reproducibility

No statistical method was used to predetermine sample size. No data were excluded from the analyses. We did not use any study design that required randomization or blinding.

### Reporting summary

Further information on research design is available in the Nature Portfolio Reporting Summary linked to this article.

## Online content

Any methods, additional references, Nature Portfolio reporting summaries, source data, extended data, supplementary information, acknowledgements, peer review information; details of author contributions and competing interests; and statements of data and code availability are available at 10.1038/s41588-023-01375-1.

## Supplementary information

Reporting Summary

Supplementary Tables Supplementary Tables 1–9.

## Data Availability

Raw sequencing data of scRNA-seq are available at the Japanese Genotype-phenotype Archive (JGA) with accession codes JGAS000593 (https://ddbj.nig.ac.jp/resource/jga-study/JGAS000593)/JGAD000722 (https://ddbj.nig.ac.jp/resource/jga-dataset/JGAD000722). A part of the raw scRNA-seq data (*n*_COVID-19_ = 30, *n*_Control_ = 31) has already been deposited^20^ and is available under controlled access at JGA with accession codes JGAS000543 (https://ddbj.nig.ac.jp/resource/jga-study/JGAS000543)/JGAD000662 (https://ddbj.nig.ac.jp/resource/jga-dataset/JGAD000662). All the raw sequencing data of scRNA-seq can also be accessed through application at the NBDC with the accession code hum0197 (https://humandbs.biosciencedbc.jp/en/hum0197-latest). Genotype data of the subjects are available at European Genome-Phenome Archive (EGA) with the accession code EGAS00001006950 (https://ega-archive.org/studies/EGAS00001006950). Raw sequencing data of scRNA-seq and genotype data are potentially identifiable and therefore under controlled access at JGA and EGA. The GWAS summary statistics of COVID-19 HGI (release 6) were obtained from https://www.covid19hg.org/results/r6/. The reference for cell type annotation of PBMC in scRNA-seq (pbmc_multimodal.h5seurat) was obtained from https://satijalab.org/seurat/articles/multimodal_reference_mapping.html.

## Code availability

The code used in the paper is available at https://github.com/REdahiro/JPN_COVID-19_scRNAseq.
