# Supplementary material for: Single-cell analyses and host genetics highlight the role of innate immune cells in COVID-19 severity
Source: Nat Genet. 2023 Apr 24;55(5):753–67. doi: 10.1038/s41588-023-01375-1 (PMC10181941; doi:10.1038/s41588-023-01375-1)
Supplement: Supplementary file 1 — Reporting Summary [file 41588_2023_1375_MOESM1_ESM.pdf]

nature portfolio

Corresponding author(s): Yukinori Okada, Atsushi Kumanogoh

Last updated by author(s): Mar 9, 2023

Reporting Summary

Nature Portfolio wishes to improve the reproducibility of the work that we publish. This form provides structure for consistency and transparency in reporting. For further information on Nature Portfolio policies, see our Editorial Policies and the Editorial Policy Checklist.

Statistics

For all statistical analyses, confirm that the following items are present in the figure legend, table legend, main text, or Methods section.

n/a	Confirmed	
☐	☒	The exact sample size (*n*) for each experimental group/condition, given as a discrete number and unit of measurement
☐	☒	A statement on whether measurements were taken from distinct samples or whether the same sample was measured repeatedly
☐	☒	The statistical test(s) used AND whether they are one- or two-sided<br>*Only common tests should be described solely by name; describe more complex techniques in the Methods section.*
☐	☒	A description of all covariates tested
☐	☒	A description of any assumptions or corrections, such as tests of normality and adjustment for multiple comparisons
☐	☒	A full description of the statistical parameters including central tendency (e.g. means) or other basic estimates (e.g. regression coefficient) AND variation (e.g. standard deviation) or associated estimates of uncertainty (e.g. confidence intervals)
☐	☒	For null hypothesis testing, the test statistic (e.g. *F*, *t*, *r*) with confidence intervals, effect sizes, degrees of freedom and *P* value noted<br>*Give P values as exact values whenever suitable.*
☒	☐	For Bayesian analysis, information on the choice of priors and Markov chain Monte Carlo settings
☒	☐	For hierarchical and complex designs, identification of the appropriate level for tests and full reporting of outcomes
☐	☒	Estimates of effect sizes (e.g. Cohen's *d*, Pearson's *r*), indicating how they were calculated

Our web collection on statistics for biologists contains articles on many of the points above.

Software and code

Policy information about availability of computer code

Data collection	No software was used in data collection.
Data analysis	Droplet libraries were processed using Cell Ranger 5.0.0 (10x Genomics). Sequencing reads were aligned with STAR (v2.7.2a) using the GRCh38 human reference genome. Filtered expression matrices generated using 'cellranger_count' were used to perform the analysis. Putative doublets were removed using Scrublet (v0.2.1) and scds (v1.10.0). Seurat (v4.1.0) was used for data scaling, transformation, clustering, dimensionality reduction and most visualization. Integration and batch correction was performed using harmony (v0.1). The final manual annotation of PBMCs was compared to the PBMC annotation of Azimuth. Differential abundance analysis was performed using Milo (v1.2.0).Differential gene expression testing was performed using edgeR (v3.32.0), and pathway enrichment analysis was performed using Clusterprofiler (v3.14.3). Spliced and unspliced transcripts were quantified using dropEst (v0.8.6). RNA velocity analysis was performed using scVelo (v0.2.3). Droplet-based sequencing data for TCR sequences and BCR sequences were analyzed using Scirpy (v0.10.0). Cell-cell interaction analysis among PBMC was performed using CellPhoneDB (v2.0.0) and NATMI (https://github.com/forrest-lab/NATMI). Gene-level association p-values from GWAS summary statistics were computed using MAGMA (v1.07), and integrating the information from scRNA-seq data with polygenic signals from COVID-19 GWAS was performed using scDRS (v1.0.1). Single-cell-level normalization was performed using scran (v1.18.5) before pseudo-bulk single-cell eQTL analysis. R statistical software (version 4.0.2) was used for the eQTL analysis of COVID-19-associated variants. SHAPEIT4 (v4.2.1) was used for haplotype phasing of genotype data. After phasing, Minimac4 (v1.0.1) was used for genome-wide genotype imputation. The code is available at https://github.com/REdahiro/JPN_COVID-19_scRNAseq.

For manuscripts utilizing custom algorithms or software that are central to the research but not yet described in published literature, software must be made available to editors and reviewers. We strongly encourage code deposition in a community repository (e.g. GitHub). See the Nature Portfolio guidelines for submitting code & software for further information.

Data

Policy information about availability of data

All manuscripts must include a data availability statement. This statement should provide the following information, where applicable:

- Accession codes, unique identifiers, or web links for publicly available datasets
- A description of any restrictions on data availability
- For clinical datasets or third party data, please ensure that the statement adheres to our policy

Raw sequencing data of scRNA-seq are available at the Japanese Genotype-phenotype Archive (JGA) with accession codes JGAS000593 (https://ddbj.nig.ac.jp/resource/jga-study/JGAS000593) /JGAD000722 (https://ddbj.nig.ac.jp/resource/jga-dataset/JGAD000722). Part of the raw scRNA-seq data (nCOVID-19 = 30, nControl = 31) has already been deposited and are available under controlled access at JGA with accession codes JGAS000543 (https://ddbj.nig.ac.jp/resource/jga-study/JGAS000543) /JGAD000662 (https://ddbj.nig.ac.jp/resource/jga-dataset/JGAD000662). All the raw sequencing data of scRNA-seq can also be accessed through application at the NBDC with the accession code hum0197 (https://humandbs.biosciencedbc.jp/en/hum0197-latest). Genotype data of the subjects are available at European Genome-Phenome Archive (EGA) with the accession code EGAS00001006950 (https://ega-archive.org/studies/EGAS00001006950). Raw sequencing data of scRNA-seq and genotype data are potentially identifiable and therefore under controlled access at JGA and EGA. The GWAS summary statistics of COVID-19 HGI (release 6) were obtained from https://www.covid19hg.org/results/r6/. The reference for cell type annotation of PBMC in scRNA-seq (pbmc_multimodal.h5seurat) was obtained from https://satijalab.org/seurat/articles/multimodal_reference_mapping.html.

Field-specific reporting

Please select the one below that is the best fit for your research. If you are not sure, read the appropriate sections before making your selection.

☒ Life sciences ☐ Behavioural & social sciences ☐ Ecological, evolutionary & environmental sciences

For a reference copy of the document with all sections, see nature.com/documents/nr-reporting-summary-flat.pdf

Life sciences study design

All studies must disclose on these points even when the disclosure is negative.

Sample size

The sample size was not predetermined and all samples that were available were processed. We recruited 73 COVID-19 cases and 75 healthy controls.

Data exclusions

Low quality cells:
Cells that had fewer than 1st percentile of UMIs or greater than 99th percentile of UMIs in each sample, as well as cells that contained greater than 10% of reads from mitochondrial genes or Hemoglobin genes, were considered low quality and removed from further analysis.

Doublets:
Cells labeled as doublets by Scrublet or scds were excluded from the analysis.

Replication

All findings were based on statistical analysis of a large patient cohort. There was no replication cohort.

Randomization

We investigated two major groups; "COVID-19 patients" and "Healthy controls".
COVID-19 patients were further categorized into groups of moderate and severe according to disease severity based on WHO guidelines.
Randomization was not applicable to this study.

Blinding

We did not apply blinding of the samples because no intervention was conducted in our study.

Reporting for specific materials, systems and methods

We require information from authors about some types of materials, experimental systems and methods used in many studies. Here, indicate whether each material, system or method listed is relevant to your study. If you are not sure if a list item applies to your research, read the appropriate section before selecting a response.

Materials & experimental systems

n/a	Involved in the study	
☒	☐	Antibodies
☒	☐	Eukaryotic cell lines
☒	☐	Palaeontology and archaeology
☒	☐	Animals and other organisms
☐	☒	Human research participants
☒	☐	Clinical data
☒	☐	Dual use research of concern

Methods

n/a	Involved in the study	
☒	☐	ChIP-seq
☒	☐	Flow cytometry
☒	☐	MRI-based neuroimaging

Human research participants

Policy information about studies involving human research participants

Population characteristics	COVID-19 patients (n = 73) are of Ease Asian ancestry, the median age was 68, 71.2% were male, and all of them were tested positive for PCR test. COVID-19 patients were further categorized into groups of moderate (n = 9) and severe (n = 64) according to disease severity based on WHO guidelines. Healthy controls (n = 75) are of Ease Asian ancestry, the median age was 34, 64.0% were male. Detailed patients characteristics are summarized in Supplementary Table 1.
Recruitment	We recruited the hospitalized cases diagnosed as COVID-19 by physicians using the clinical manifestation and PCR test results. We included all COVID-19 cases treated at Osaka University from July 2020 to September 2021 who gave consent for the study. Thus, there is little selection bias. All control participants were recruited at Osaka University.
Ethics oversight	This study was approved by the ethical committee of Osaka University Graduate School of Medicine.

Note that full information on the approval of the study protocol must also be provided in the manuscript.
